# Sexual Dimorphism in Colon Cancer

**DOI:** 10.3389/fonc.2020.607909

**Published:** 2020-12-09

**Authors:** Maria Abancens, Viviana Bustos, Harry Harvey, Jean McBryan, Brian J. Harvey

**Affiliations:** ^1^Department of Molecular Medicine, RCSI University of Medicine and Health Sciences, Beaumont Hospital, Dublin, Ireland; ^2^Department of Surgery, RCSI University of Medicine and Health Sciences, Beaumont Hospital, Dublin, Ireland; ^3^Departamento de Acuicultura y Recursos Agroalimentarios, Programa Fitogen, Universidad de Los Lagos, Osorno, Chile; ^4^Department of Medical Oncology, Cork University Hospital, Cork, Ireland; ^5^Centro de Estudios Cientificos CECs, Valdivia, Chile

**Keywords:** sexual dimorphism, colon cancer, estrogen, Kv channels, Wnt/β-catenin, GPER

## Abstract

A higher incidence of colorectal cancer (CRC) is found in males compared to females. Young women (18–44 years) with CRC have a better survival outcome compared to men of the same age or compared to older women (over 50 years), indicating a global incidence of sexual dimorphism in CRC rates and survival. This suggests a protective role for the sex steroid hormone estrogen in CRC development. Key proliferative pathways in CRC tumorigenesis exhibit sexual dimorphism, which confer better survival in females through estrogen regulated genes and cell signaling. Estrogen regulates the activity of a class of Kv channels (KCNQ1:KCNE3), which control fundamental ion transport functions of the colon and epithelial mesenchymal transition through bi-directional interactions with the Wnt/β-catenin signalling pathway. Estrogen also modulates CRC proliferative responses in hypoxia *via* the novel membrane estrogen receptor GPER and HIF1A and VEGF signaling. Here we critically review recent clinical and molecular insights into sexual dimorphism of CRC biology modulated by the tumor microenvironment, estrogen, Wnt/β-catenin signalling, ion channels, and X-linked genes.

## Introduction

**Figure 1:**
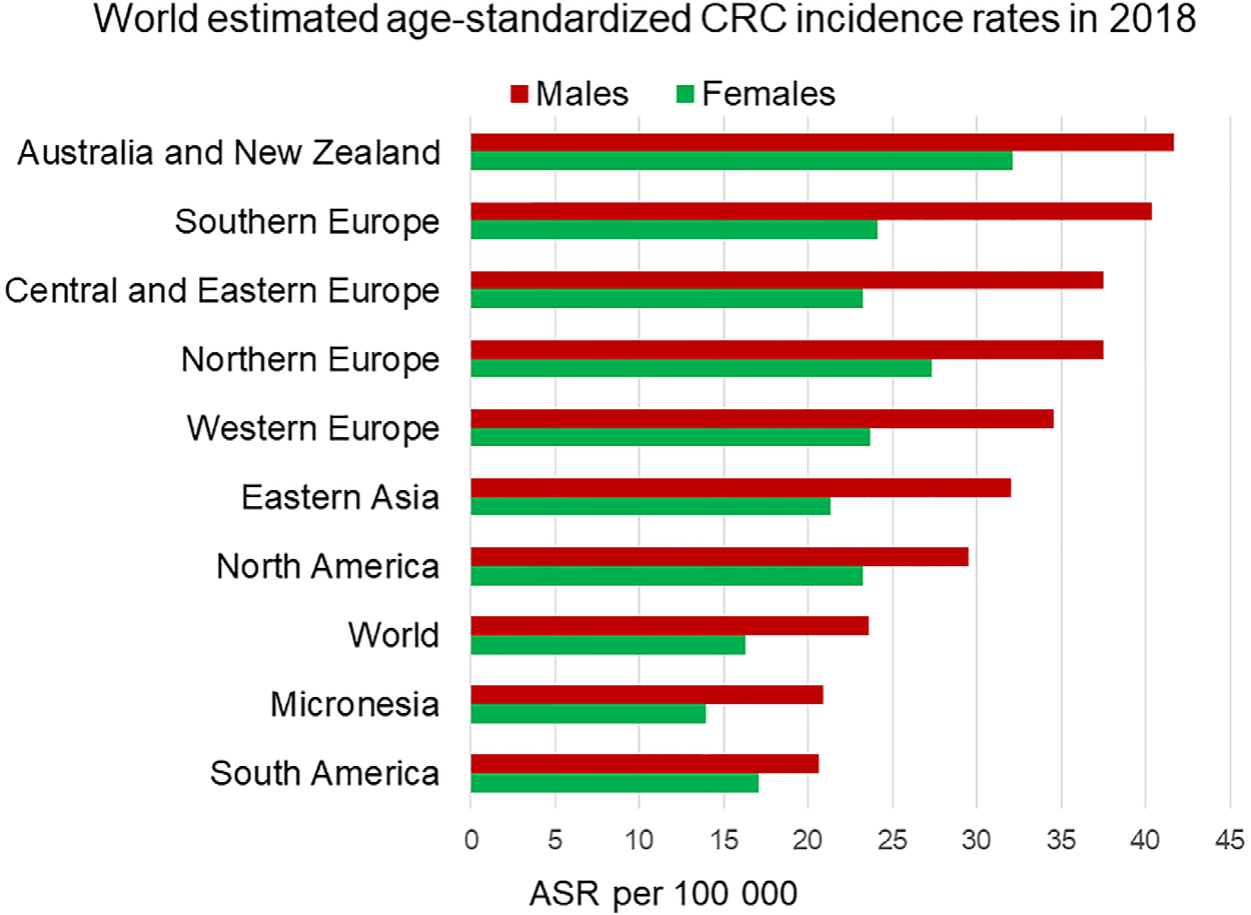
Estimated age-standardized incidence rates worldwide of colorectal cancer in 2018. Men (in red) display higher incidence rates than age-matched women (in green) in each region. ASR, Age-standardised rate. Data from the Global Cancer Observatory https://gco.iarc.fr/today/home (5).

When describing sex differences in cancer it is important to discriminate between sexual dimorphism (biological differences in hormones and genes) and gender differences (non-biological differences in societal attitudes and behaviour). In this review we expressly examine sexual dimorphism in colorectal cancer (CRC); physiological or pathophysiological characteristics arising from differences in hormonal or other biological parameters such as genetic inheritance and epidemiology of underlying biological causes. This should not be confused with any apparent ‘gender gap’ in CRC; differences in the incidence of CRC between women and men that can be attributed to conditions other than biological as reflected in societal, political, cultural, economic or behaviour such as nutrition, hygiene or access to screening and health care. We acknowledge the non-binary nature of gender but appreciate that most studies in this field have used dichotomisation to aid analysis and interpretation of sometimes very complex data. In this review, we will treat sexual dimorphism of CRC in detail and, for completeness, also include a brief review of underlying causes of a non-biological gender gap in CRC. Colorectal cancer is the third most common cancer and the second leading cause of cancer-related deaths worldwide (1). There is a large body of literature on sexual dimorphism in a wide number of cancers, the majority of which show male predominance with notable exceptions of breast and thyroid cancer, which are female-dominant (2, 3). CRC rates vary markedly around the world due to genetic and lifestyle associated risks and the availability of population-screening programmes for early detection. A clear sexual dimorphism in age-standardised incidence rates of CRC has been observed in every region of the world (4), with lower incidence in females compared to males (**Figure 1**).

**Figure 2:**
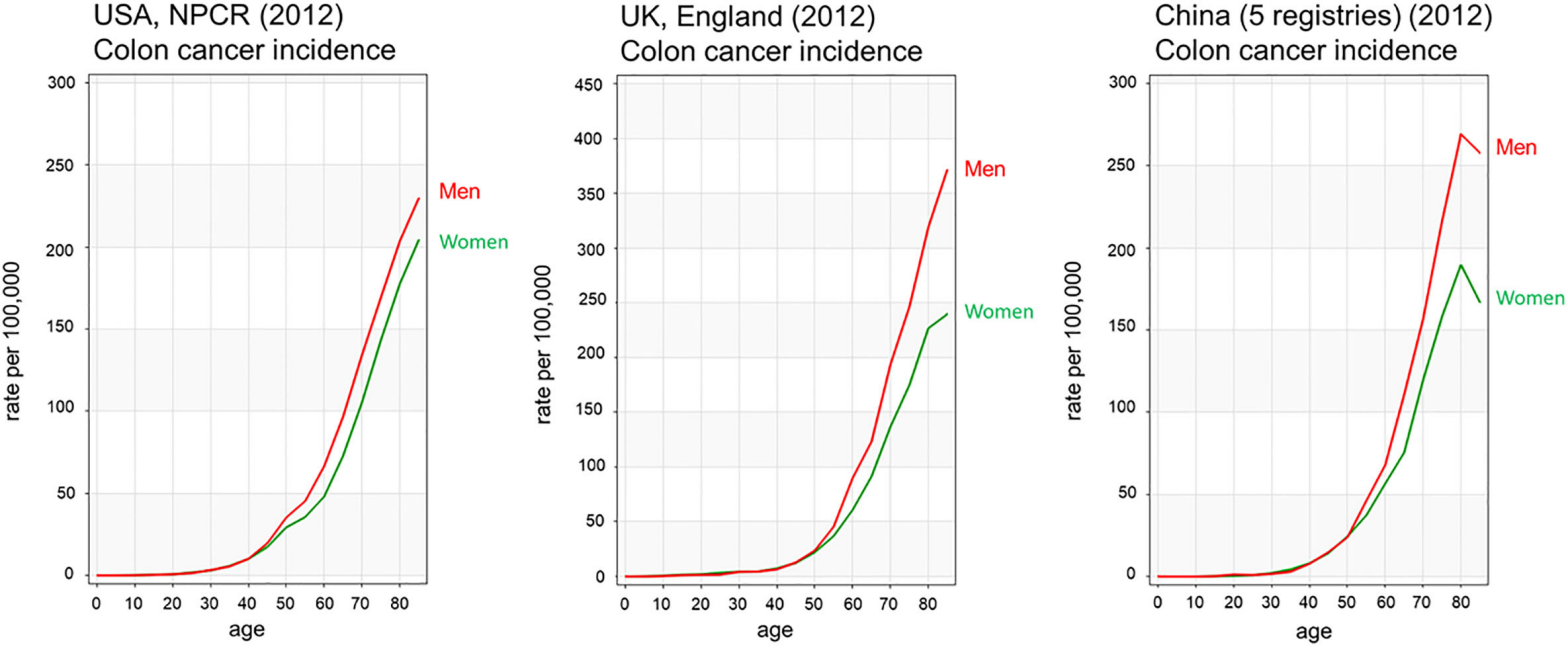
Age-standardised colorectal cancer incidence rates of men and women in USA, UK and China. Colon cancer incidence rates by age show men have higher incidence rates than females from 45 years of age Colon cancer is shown in red for men and green for women. Data from Ci5plus in the International Agency for Research on Cancer (6). https://ci5.iarc.fr/CI5plus/Pages/graph1_sel.aspx.

**Figure 3:**
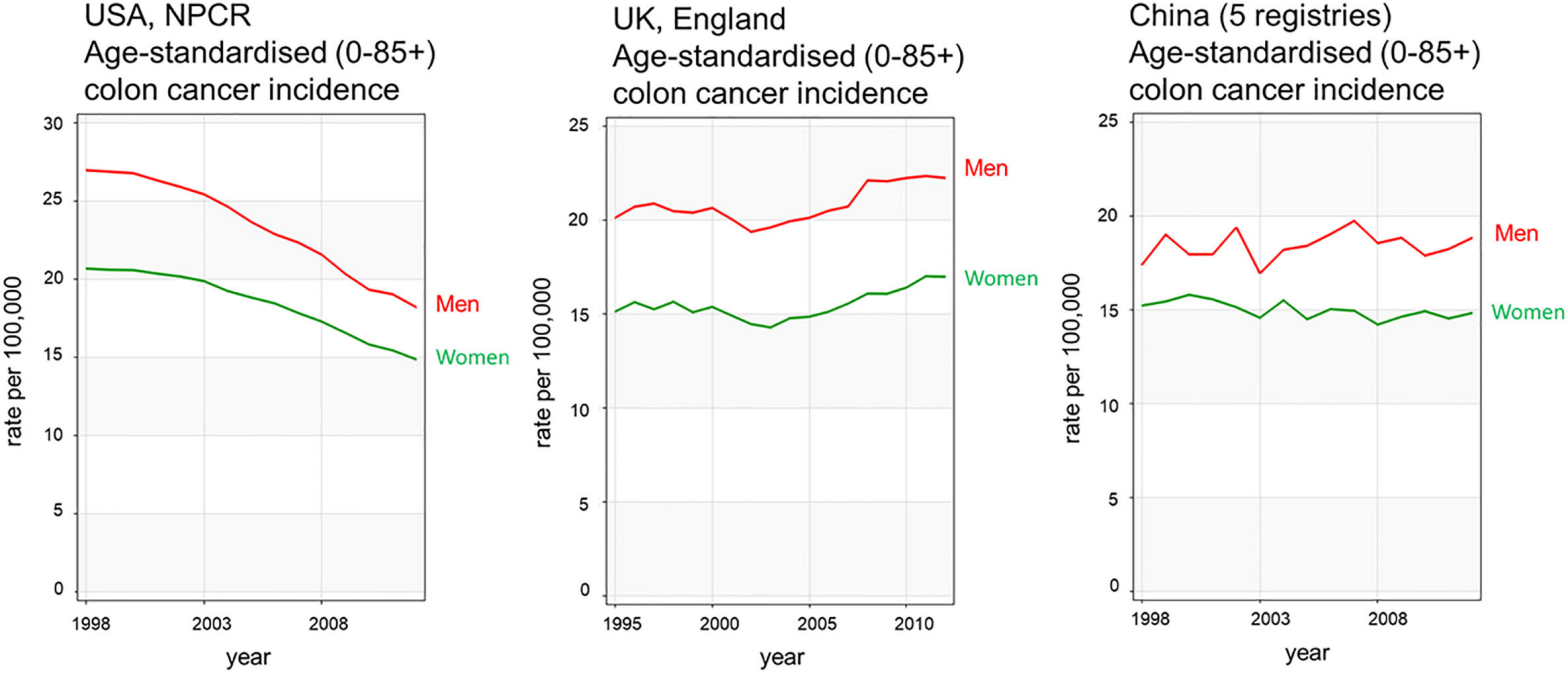
CRC incidence rates over time show sexual dimorphism and have been relatively stable during the 25 years examined. Examples from United States, United Kingdom, and China colon cancer registries are shown in red for men and green for women. Data acquired from Ci5plus in the International Agency for Research on Cancer (6) https://ci5.iarc.fr/CI5plus/Pages/graph4_sel.aspx.

According to the Global Cancer Observatory (5), the age-standardized incidence rate in men is 45% higher (23.6 per 100,000 person-year) compared to women (16.3 per 100,000 person-year). Also, men have a 50% higher cumulative risk (CR) to develop CRC than women (CR 2.75 vs. 1.83). Looking at different age-frames, CRC incidence rates under 50 years old are very low and there is not a clear sex disparity. In every age-group above 50, women have lower incidence rates than age-matched males (6). This effectively results in 4–8 years delay in women compared to men, such that women aged 65 for example, have similar CRC incidence to men aged 60 (7). Sex hormones are an obvious difference between men and women and, as we will discuss, can impact cancer initiation and progression by multiple mechanisms. With the majority of women in the post-menopausal stage at CRC diagnosis, the sexual dimorphism in more elderly CRC incidence rates may not entirely be due to sex hormones unless these have a protective effect during child-bearing years to delay the onset of CRC. There is still much debate how sex hormones, which are known to be carcinogenic in a wide number of tissues may exert protective effects in the colon (8, 9). In spite of the great variability in CRC incidence rates from one region of the world to the next, sexual dimorphism in colon cancer incidence rates is consistently observed for countries in Europe, the Americas and Asia (**Figure 2**). Moreover, the difference in CRC incidence between men and women is not a new phenomenon and has been observed steadily for at least the past 25 years (**Figure 3**). Thus, there is a well-established sexual dimorphism in CRC throughout the world with men displaying higher incidence rates of CRC compared to women.

**Figure 4:**
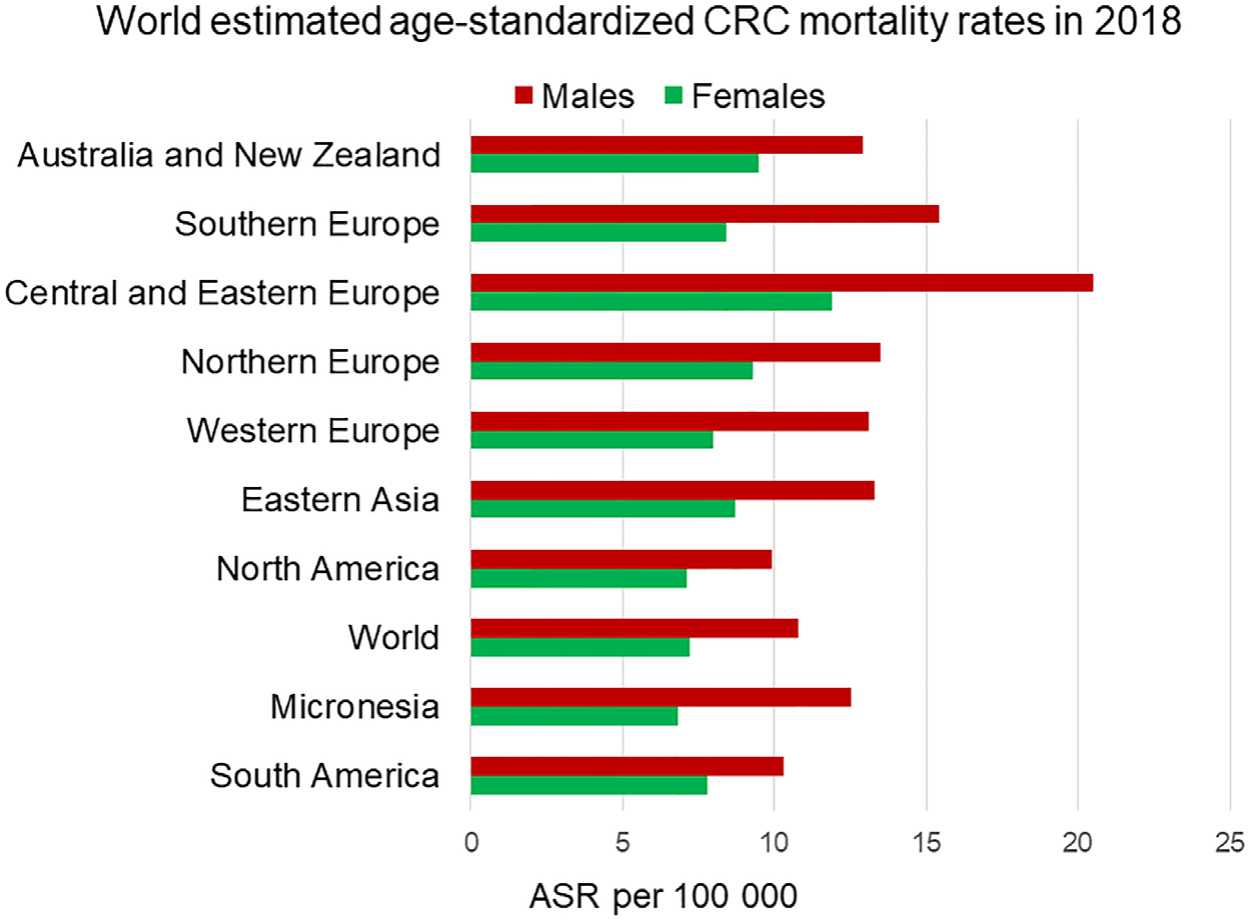
Estimated age-standardized mortality rate worldwide of colorectal cancer in 2018. Men (in red) exhibit higher mortality rates than female (in green) in every region. ASR, Age-standardised rate. Data from the Global Cancer Observatory (5).

Mortality rates from CRC are also higher in men compared to women and this is consistently seen across different regions of the world (**Figure 4**). The age-standardized mortality rate for men is 50% higher (10.8 per 100,000 person-year) than for women (7.2 per 100,000 person-year) (1). It is challenging to determine how much of the sexual dimorphism in CRC mortality is attributable to the sex differences in CRC incidence rates. Several retrospective studies have shown female CRC patients to have longer survival rates than male patients. For example, a German population-based cohort study including 185,967 patients showed women had significantly better overall (HR 0.853) and recurrence-free survival (HR 0.857) than men (10). A meta-analysis from 2017 including 37 clinical trials determined that women had better overall (HR 0.87) and cancer-specific survival (HR 0.92) than men (11). In the same way, the EUROCARE-4 study that analyzed data of patients diagnosed between 1995 and 1999 from 23 European countries showed women had a 2.2% advantage in 5-year average and region-adjusted survival for colon and rectal cancer (12). The most recent EUROCARE-5 study, which evaluated patients from 29 European countries diagnosed between 2000 and 2007 showed the advantage for women in colon cancer survival was insignificant (13). Indeed, some studies have failed to detect any survival benefit for women and attribute the higher CRC mortality rates in men to higher incidence rates. A cross-sectional study from the UK including 164,980 CRC patients, showed no significant age-standardized survival benefit for women compared to men (14). This lack of consensus could be due to a confounding reproductive hormonal effect in survival of women strongly dependent on age. Generally, the benefit in CRC survival, which has been attributable to sexual dimorphism, has been associated with the pre-menopause stage in women. Multiple studies have reported that pre-menopausal patients have better 5-year survival rates than age-matched male patients, and younger women (18–44 years) show lower mortality over comparable time frames compared to older women (over 50 years) (15, 16). In contrast, CRC female patients over 65 years old show worse survival rates than age-matched male patients. They tend to be diagnosed in a more advanced stage than men and have a more aggressive cancer type (17, 18). Indeed, it has been suggested that lower survival rates in older women could be due to being diagnosed at a more advanced stage than men (19, 20).

Sex differences exist regarding the type of tumor, which is diagnosed. Women show a higher frequency of right-sided tumors than men (21). Right-sided proximal tumors occur predominantly in women and older patients, and are less common than left-sided distal tumors. In addition to the location, right and left-sided tumors have been shown to differ in several clinicopathological features such as immune infiltration, differentiation or microsatellite instability status (MSI). High-MSI tumors comprise close to 15% of all CRC cases, and they locate predominantly in the proximal colon (22). MSI status has been shown to impact sensitivity to CRC treatments and prognosis. For example, immunotherapy has been shown to improve prognosis, particularly of MSI-high tumor carrying patients, whereas anti-EGFR and 5-FU adjuvant therapies exert little and no benefit to MSI-H tumors in contrast to MSS tumors (23). In terms of survival, metastatic CRC patients with right-sided primary tumors have worse survival rates compared to left-sided tumors (24). This may be a confounding feature in our interpretation of mortality rates. Right-sided tumors are more difficult to diagnose by colonoscopy because of their flat shape neoplasia compared to the polyp type neoplasia from left-sided tumors (25).

Overall, sexual dimorphism exists at multiple levels in CRC; women have a lower risk to develop CRC than men. Females at a younger age are less likely to die from CRC than age-matched male patients and certain types of CRC occur predominantly in women. Understanding the molecular mechanisms underlying this sexual dimorphism and how women are protected against CRC should guide future treatment and therapy developments.

## Sexual Dimorphism of Hormones and Receptors in CRC

The bulk of the studies cited above suggest a protective role for the sex steroid hormones in CRC development. The role of reproductive hormones and their receptors in the onset and progression of CRC is not well understood and is still surprisingly under-researched. On balance, estrogen appears to be the major hormone underpinning sexual dimorphism in CRC. There are some leads that show promise in understanding the molecular mechanisms for protective effects of estrogen against the onset and development of CRC tumors. As we shall discuss in detail, some of these targets include pivotal estrogen-regulated cell signaling pathways including the Wnt/β-catenin pathway, ion channels acting as tumor suppressors such as the Kv channel KCNQ1, and membrane estrogen receptors such as GPER.

### Sex Hormones

One obvious biological distinction between the sexes is the difference in the levels of circulating sex hormones. All sex steroid hormones derive from cholesterol and are classified into progestogens (progesterone), androgens (testosterone), and estrogens (17β-estradiol, estriol, and estrone). Estrogens are synthetized from androgens, which in turn derive from progestogens produced from cholesterol. Sex hormones are produced in the testis (men) and ovaries (women), but also in the adipose tissue, adrenal glands, brain, skin, and bone. Adipose tissue becomes an important source of estrogens in postmenopausal women and obese men. Higher Body Mass Index (BMI) and lower physical activity levels have been positively associated with higher levels of circulating estrogens in postmenopausal women and men (26, 27). Sex hormones are mainly bound in the plasma to albumin and to sex hormone-binding globulin (SHBG). Only 1–3% of circulating sex hormones is unbound and this fraction is considered to be the most biologically active. Measuring the concentration of free hormones becomes a technical challenge due to very low concentrations. Thus, very often this fraction is calculated from both SHBG and total hormone concentration according to reference mathematical models. Both sexes produce the same sex hormones with differential effects depending on circulating concentration, receptor expression and hormone-specific interactions with target organs. Normal estrogen levels in premenopausal adult females range between 15–350 pg/ml and the amount of estrogen depends on the phase of the menstrual cycle, ranging from 60–250 pg/ml in the proliferative phase and 75–450 pg/ml in the luteal phase. Estrogen levels in postmenopausal women typically fall below 10 pg/ml and are similar to levels in age-matched males (28). HRT can raise estrogen to levels of 50–100 pg/ml.

Epidemiological observations, particularly in women on hormone replacement therapy (HRT) and *in vitro* studies support a role for the female sex hormones in protecting against colorectal cancer (29, 30). There have been some attempts to associate circulating levels of endogenous estrogens and CRC risk. Still, the data so far is limited to postmenopausal women and shows mixed outcomes. Studies of the effects of HRT on cancer progression in postmenopausal women attributed a protective role to female sex hormones and encouraged many researchers into the area (31). Similarly, oral contraceptive use has been reported to decrease CRC risk by 10–20% (32). Consistent with these findings, oophorectomy and the early suppression of female sex hormones has been shown to increase CRC risk by 30% (33). The Women´s Health Initiative (WHI) Study is perhaps the most cited study linking HRT with CRC protection. The WHI was a double-blind randomized clinical trial performed in the early 2000s and showed combined therapy of estrogen plus synthetic progesterone resulted in a 38% reduction in colon cancer, although the protection did not last in the follow-up years of the study. Postmenopausal women with prior hysterectomy given an estrogen alone therapy did not benefit from any protection (34). The WHI study was far from perfect. Indeed, the average age of women enrolled in the study was 63 years, which indicates they started HRT many years after the menopause, which could impact the effect of sex hormones. At this stage there is compelling evidence from multiple studies of HRT in reducing CRC risk suggesting that the delay, duration, HRT formulation, and time since menopause are key elements impacting the strength of CRC protection. Reproductive factors used as surrogate markers for lifetime estrogen exposure such as parity, age of birth of first child, age of menarche, and age at menopause are also confounding factors in interpreting HRT data on sex-bias in CRC. Combination estrogen, progesterone, and HRT were studied in a 2012 meta-analysis based on four randomized trials, 8 cohort studies and 8 case-control studies included data of postmenopausal women collected from the EU, Canada, and the United States between 1965 and 2006 and had a follow up of 3–15 years. Results from this meta-analysis show that both current estrogen alone and current estrogen plus progesterone therapy reduced colon cancer risk by 30% (35). Recent studies are focused on assessing HRT effect on different CRC molecular subtypes. As an example, a 2018 meta-analysis on CRC risk by microsatellite instability status concluded that HRT reduced CRC risk in microsatellite stable (MSS) but not in highly unstable (MSI-H) tumors (36).

HRT has also been shown to reduce mortality in CRC female patients. A 2019 meta-analysis including 4 USA and 1 Swedish cohort has reported current but not former HRT use reduced colon cancer-specific (hazard ratio of 0.71 [95% confidence interval (CI), 0.62–0.80]) and overall (hazard ratio of 0.74 [95% CI, 0.67–0.81]) mortality (37). These findings are consistent with earlier data from the PLCO Cancer Screening randomized trial run from 1993–2001 in the USA, which showed HRT current-users had lower cancer-specific (hazard ratio 0.63 [95% CI, 0.47–0.85]) and overall (hazard ratio of 0.76 [95% CI, 0.72–0.80]) mortality compared to never-users (38). The similar benefit conferred by either estrogen alone or HRT in many studies point to estrogen as the main player for protection in CRC.

A small number of studies have evaluated cancer risk in the intersex spectrum population. Given the higher estrogen levels in men with Klinefelter syndrome and conversely, the reduced estrogen levels in Turner syndrome women compared to karyotypically normal men and women, they become a valuable sample to evaluate the role of sex hormones in CRC. The available data suggest that men with Klinefelter syndrome have decreased risk for CRC (39), whereas Turner syndrome women have an increased risk comparing to women (40), backing the idea of estrogen as a protective factor against CRC. However, the numbers are too small to get a significance and the results should be further validated. Transgender population have also been of interest to evaluate sex-hormone-driven cancers but there is at present a lack of information regarding CRC incidence in this group of patients (41).

Besides the systemic estrogen levels, the local intra-tissue expression of estrogen and sex steroid converting enzymes can also modulate CRC progression in a sex-specific manner. One study showed significant association between estrogen (E1 estrone and E2 17β−estradiol) on CRC patients’ clinical outcome with high intratumoral concentrations of total estrogen significantly associated with adverse clinical outcome (42). This study also showed that patients negative for steroid sulfatase and positive for estrogen sulfotransferase had significantly longer survival. Steroid sulfatase promotes the synthesis of E1, whereas estrogen sulfotransferase inactivates E1 thus supporting a prognostic role for these enzymes and E1 in CRC. However, another study failed to find significant correlation between tumoral tissue concentrations of estrogen and CRC survival (43). Instead patients with low intra-tissue concentration of estrone coupled with low estrogen receptor ESR1 expression showed increased CRC recurrence compared to patients with high *ESR1* mRNA levels and high E1 concentration. It would appear that estrogen effects on CRC patient overall and disease-free survival depends on the expression levels of estrogen receptor and possibly on the use of oral contraceptive or HRT in these patient groups. Extragonadal production of the active estrogen 17β−estradiol may occur by the aromatase pathway from androstenedione or the sulfatase pathway from estrone sulfate followed by E1 reduction to E2 by 17-β-hydroxysteroid dehydrogenase (HSD17B1).Thus *HSD17B1* gene expression may play an important role in the production of E2 in the colon and modulate the role of endogenous E2 in the prevention of carcinogenesis. In support of this possibility, a significant decrease in HSD17B1 transcript and protein levels have been reported in (CRC) from the proximal colon (44).

Progesterone has also been considered as a potential contributor to CRC protection. In general, normal serum progesterone levels are lower in males than females, however males have similar progesterone levels (~1 ng/ml) as postmenopausal women, and as women at the beginning of their menstrual cycle. Progesterone levels increase in women in the middle of the menstrual cycle from 5 to 20 ng/ml, and in pregnancy from 11 to 90 ng/ml (28). The potential role of progesterone in CRC is interesting since this hormone is cyclically elevated in the early stages of the disease in women of childbearing age. Moreover, the number of postmenopausal women using natural progesterone instead of synthetic progestins in hormone replacement therapy is increasing. There is some recent evidence implicating progesterone in reducing CRC but this seems to be additive to estrogen signaling *via* the estrogen receptor ERβ (45). A retrospective clinical trial found no evidence for association of progesterone receptor (PGR) expression with CRC survival outcomes (46). In addition, data from CRC animal models show no effect on polyp number and size of progesterone or medroxyprogesterone acetate (MPA) in ovariectomized rats (47). Also, the systemic absence of progesterone receptor did not impact colonic polyp formation in the PRKO-Apc^Min/+^ mice. MPA has been reported to reduce adenoma incidence in the CRC and menopause model AOM-VCD-mice, suggesting a protective role for progesterone specific to postmenopausal incidence of CRC (48).

Testosterone levels are much higher in men than women. Men have been reported to carry 5–20 ng/dl of free circulating testosterone, reaching their peak at the age of 20 and decreasing the levels gradually from the age of 30–40; in contrast to 0.1–0.5 ng/dl in age-matched women. Total testosterone levels are reported in the range of 220–850 ng/dl in men and 1.7–48 ng/dl in women (49). Female testosterone levels decrease slightly from age 40 years and further in postmenopausal women and are much lower than age-matched males (50). Could the lower testosterone levels in women be contributing to CRC risk? The evidence is far from clear. Some studies have used animal models to test the effect of testosterone on CRC tumorigenesis. The APC^PIR/+^ rat harbours an *Apc* non-sense mutation in heterozygosis that leads to spontaneous colonic tumors. These tumors occur in greater numbers in male compared to female rats, mimicking the human sexual dimorphism. Males also develop a larger number of adenomas in the AOM mouse in, which the mutagenic agent azoxymethane induces spontaneous colonic carcinogenesis. Castration decreased the number of developed colonic adenomas in both the APC^PIR/+^ rat and in the AOM mouse models (51). In turn, implantation of dihydrotestosterone in the APC ^PIR/+^ rat and testosterone supplementation in the AOM mouse, along with castration, increased the number of adenomas. The combined studies in these animal models point to a tumor promoting role of testosterone. Consistent with these observations, a recent study in postmenopausal women in Japan (52) including 185 CRC patients and 361 controls, showed that higher testosterone levels were significantly associated with CRC risk (odds ratio 2.1 [95% CI, 1.11–3.99]). Lower androgenicity in men, as a result of reduced androgen receptor activity by hypermethylation or lower circulating dehydroepiandrosterone sulfate, has been associated increased CRC risk (53, 54). A recent USA study including 732 CRC patients and 1156 controls from 4 prospective cohorts reported that high testosterone levels in men were significantly associated with lower relative-risk for CRC (highest vs. lowest quartile 0.65) and showed no association in postmenopausal women (55). Also, a prospective study evaluating 107,859 men diagnosed with prostate cancer from the American SEER-Medicare database has shown orchidectomy and long-term androgen-deprivation therapy (more than 25 months) were significantly associated with higher CRC risk (hazard ratio of 1.37 [95% CI, 1.14–1.66] and 1.31 [95% CI, 1.12–1.53] respectively) (56). These are of course epidemiological observational studies and not case-controlled interventional trials, thus there may be many confounding factors leading to the different CRC risk associations in different populations. Expression of the androgen receptor has also been explored for association with CRC risk. In a case-control study including 550 CRC patients and 540 controls, longer CAG repeats in the androgen receptor gene (AR) that reduce the transcription rates, have been associated with higher CRC risk and lower 5-year median overall survival (hazard ratio of 1.4 [95% CI, 1.04–1.79]) in CRC patients (57). In contrast, a German population-based study of 1,798 CRC patients and 1,810 controls reported no association between CAG repeats in AR and CRC prognosis (58). Recently, a study of the UK CRC Biobank concluded that a small fraction of the positive association between obesity and CRC risk in men is attributable to a decrease in SHBG and testosterone (59). Lastly, adding to this complexity, some smaller prospective population-based studies failed to detect any association of testosterone with CRC risk (60, 61). In summary, the role of testosterone in CRC is not yet clear, but some of the compounding factors are age-dependent differential effects of the hormone, tumor stage and tissue environment factors such as hypoxia.

### Estrogen Receptors

Estrogen produces biological effects in target tissues though its binding to selective estrogen receptors of various sub-types. The tissue-specific and subcellular expression of estrogen receptor isoforms confer selectivity of action of estrogen to initiate distinct cell signaling pathways and nuclear responses. Many steroid hormones including estrogen produce both rapid and latent biological actions, which can be divided into membrane-initiated non-genomic effects (seconds – minutes) and nuclear genomic responses (hours-days) (62). Genomic responses mainly involve estrogen binding to the major wild-type estrogen isoforms ERα (encoded by *ESR1*) and ERβ (encoded by *ESR2*) in the cytosol (with release from heat shock proteins) or directly in the nucleus to produce receptor dimerization and interact with cofactors to induce gene transcription and protein synthesis. Non-genomic rapid responses to steroid hormones (RRSH) on the other hand involve estrogen binding to a membrane-associated estrogen receptor followed usually by transactivation of other membrane receptors (G-proteins or EGFR) to induce rapid protein kinase signaling, which modulate cellular processes such as calcium signaling, pH, ion channel activity and metabolic pathways (for a recent review and listing of RRSH publications see (63). The receptors involved in transducing rapid non-genomic responses to estrogen are the membrane estrogen receptor mER (shown to be a palmitoylated full-length 66 KDa ERα) and the G protein coupled estrogen receptor (GPER, encoded by the *GPER1* gene). Most of the rapid actions of estrogen have been shown to be transduced by mER (64), whereas GPER appears to become biologically important as an estrogen receptor in tissues where the expression of ER isoforms is low or undetectable (65). It is important to note that a strict dichotomy does not appear to exist between rapid non-genomic and latent genomic estrogen actions. Significant biologically relevant cross-talk has been demonstrated between genomic and non-genomic signaling pathways (66). The distinct physiological actions of extranuclear and nuclear estrogen receptors have been elucidated using genetically modified mouse models of MOER membrane-only estrogen receptor (67), NOER nuclear-only estrogen receptor (68) and ERKO estrogen receptor null (69). Membrane initiated rapid responses to estrogen have been shown to influence the latent genomic responses (for example by MAPK phosphorylation of the ligand-receptor complex to facilitate nuclear translocation and phosphorylation of transcription factor signaling such as PKA-CREB) and vice versa genomic effects can regulate rapid responses through the synthesis of protein kinases essential for rapid actions in the cytosol. Truncated forms of ERs have been found to be expressed mainly in the membrane and cytosol of cancer cells. These splice variants of ER comprise mainly of 46- and 36-kDa ERα and may modulate non-genomic membrane-initiated cell signaling of proliferative pathways and genomic nuclear regulation of wild-type ERs in cancer cells (70). The various roles of non-genomic and genomic actions of ER isoforms and GPER in CRC development are discussed in detail below.

Given the preponderance for a lower incidence of CRC in pre-menopausal women, the weight of clinical and experimental evidence points to estrogen as the major sex steroid hormone underlying sexual dimorphism in CRC. The protective role of estrogen in the female against colorectal cancer has been mainly associated with expression levels of the ERβ in intestinal epithelial cells (71, 72). The ERβ1, ERβ2, and ERβ5 isoforms are expressed in the colon with variable levels along the crypt axis (73) and are reduced in tumor cells compared to normal colon (74, 75). However, the reduction of ERα splice variants ERα46 and ERα36 mRNA levels have also been observed in colon cancer tissue compared to normal matched controls (76). Experiments in ERβ KO mice indicated an accelerated migration of cells from crypt base to apex, while apoptosis and cell differentiation were reduced (77). The experimental overexpression of ERβ in colon cancer cells revealed that ERβ inhibits proliferation by blocking of the cell cycle in G1-S phase (78) possibly involving repression of mir-17 (79), and stimulating apoptosis (80). Moreover, in addition to anti-tumorigenic actions, ERβ induces anti-inflammatory signaling in CRC cells (81). Other protective mechanisms attributed to ERβ in the colon include upregulation of tight junction proteins occludin (OCLN) and JAMA (F11R) to preserve homeostasis of paracellular permeability (82). Estrogen may thus have a protective role in CRC *via* signal transduction through full-length ERβ (ERβ1) expression (83). ERβ1 is the most highly expressed estrogen receptor in the healthy colonic epithelium and the expression of ERβ1 is reduced in tumor cells compared to normal colon (84, 85). Sustained ERβ1 expression in CRC correlates with better prognosis and patient survival, whereas loss of ERβ1 expression is correlated with worse prognosis (86). Estrogen has been shown to increase ERβ levels and this is a likely mechanism by which estrogen confers protection against CRC (87). These findings, however, do not explain potential anti-proliferative effects of estrogen in advanced tumors or whether estrogen can promote tumor progression following the loss of ERβ. The possibility that estrogen signaling through alternate estrogen receptors confers protection is an attractive hypothesis but this premise remains controversial.

Perhaps the most convincing evidence to date for protective estrogen activity in CRC in the absence of ER concerns the role of membrane-bound G protein-coupled estrogen receptor GPER (88, 89). Several studies have demonstrated diverse functions of GPER in the colon, including the regulation of visceral hypersensitivity and gut motility, immune responses in inflammatory bowel diseases (90, 91), and the modulation of cell migration and proliferation in CRC cell lines (92). The effect of GPER to stimulate gut motility is interesting in that CRC neoplasms have been directly linked to chronic constipation (93). Mucosal inflammation is also a risk factor in the development of CRC neoplasms (94, 95) and a sex-dependent regulation of GPER expression and signal transduction has been proposed in inflammatory bowel disease (96). Disrupted expression of estrogen receptors in the intestinal mucosa of patients with Crohn’s disease and ulcerative colitis indicates that estrogen signaling may be important to maintain epithelial homeostasis and the local immune response in a sex- and age-dependent manner (97). Secretory diarrhoea is a major cause of mucosal inflammation and estrogen has been demonstrated to produce a rapid non-genomic anti-secretory response in colon, which is both female sex-dependent (not observed in males) and estrous cycle-reliant in its potency (98, 99). Thus estrogen and GPER modulate key physiological functions in the intestine, which when dysregulated can predispose to CRC neoplasm development.

The evidence so far indicates that estrogen effects in advanced CRC tumors may be transduced *via* GPER given the relative absence of expression of ERα or ERβ in CRC patients and cell lines studied. GPER has been described as a tumor promoter in certain cancers such as breast cancer *via* activation of EGFR, STAT5 and MAPK/extracellular regulated kinase (ERK) pathways (100). In CRC, the expression of GPER is reported to act variously as a tumor suppressor or promoter depending on the stage of the disease and expression levels of ER and GPER (101). For example, in 2017, one published study demonstrated that GPER activation by its specific agonist G-1 in CRC cell lines (HCT116 and SW480 cells) inhibited cell growth and promoted tumor cell apoptosis (102). However, a separate study in the same year showed that estrogen can accelerate the proliferation of CRC cell lines expressing GPER (including HCT116 cells) but not cells expressing ER, *via* a positive feed-back loop of GPER induced expression of steroid sulfatase, which activates conjugated estrogen, further enhancing cancer progression (103). In two recent cohort studies and three independent investigations from the Oncomine database (104), it was shown that the expression of *GPER* was significantly decreased with increasing stage and lymph node metastasis of CRC patients. As colon cancer progresses to advanced stage disease, *GPER* expression was found to be greatly reduced in cancerous tissue compared to adjacent healthy colon and low GPER expression was associated with reduced survival (105). More recently, a Kaplan-Meier analysis of mRNA expression data, found high *GPER* expression to be associated with poor relapse-free survival in women with stage 3/4 (but not stage 1/2) CRC while there was no correlation of *GPER* expression in men with disease of any stage (106). Thus it would appear that the role of GPER in CRC is both sexually dimorphic and dependent on the stage of the disease.

Although multiple lines of evidence suggest that GPER may play an important role in colorectal carcinogenesis, the interpretation is complicated by sexual dimorphism, stage of disease and the divergent roles of GPER in clinical CRC patient studies and CRC cell lines. Is there an unambiguous explanation for these discrepancies? Possible answers lie within the tumor microenvironment where metabolic signals may modulate the signaling pathways regulated by estrogen/GPER.

## Sexual Dimorphism and the Tumor Microenvironment in CRC

### Hypoxia

**Figure 5:**
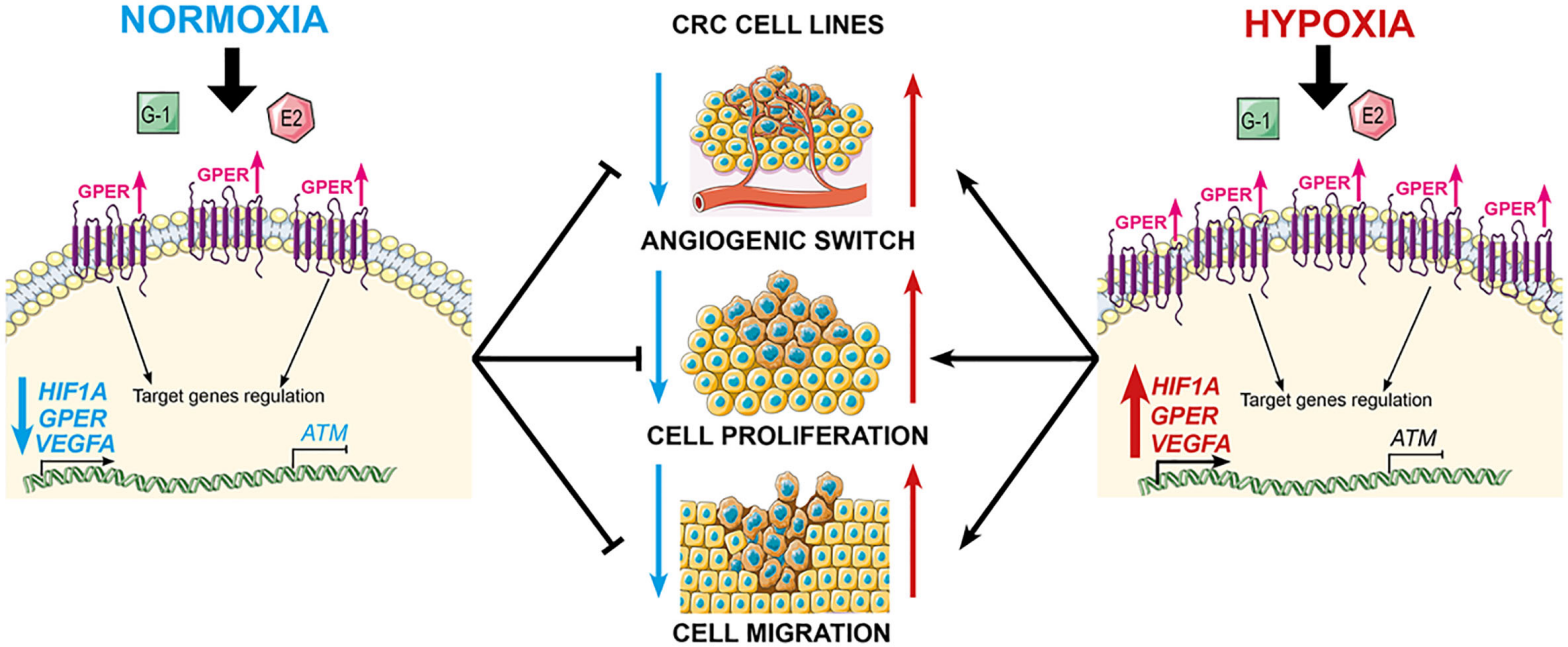
GPER mediates opposing functions of estrogen in CRC cells, depending on oxygen levels. GPER agonists suppress cell proliferation and migration under normoxic conditions, but stimulate proliferation under hypoxic conditions. Estrogen synergises with hypoxia by repressing *ATM* expression *via* GPER, and combined with the activation of HIF1A and VEGFA, potentiates the hypoxia-induced cell proliferation and migration.

The influence of the tumor microenvironment, in particular hypoxia, on both neoplastic transformation and tumor progression is well known (107). Hypoxia (≤3% oxygen) and anoxia (≤0.3% oxygen) are potent drivers of cancer cell proliferation, angiogenesis, metastatic potential and resistance to chemo- and radio-therapy (108). The induction and activation of the hypoxia-inducible transcription factor HIF1A and the HIF target of vascular endothelial growth factor alpha (VEGFA) are separately associated with poor CRC clinical outcome (109, 110). Functional cross-talk between GPER activation and EGF signalling has been demonstrated in CRC ER-negative cell lines and modulates the potential for CRC cell proliferation and migration (111). Comparative studies of estrogen effects on GPER signaling in normoxia and hypoxia in CRC cell lines revealed opposing effects of estrogen on HIF1A and VEGFA depending on ambient oxygen levels (106). Under normoxic conditions, estrogen and GPER agonists suppressed both HIF1A and VEGFA expression thus reducing the cell signals for cell proliferation and growth in the HT29 CRC cell line model. In contrast under hypoxic conditions, estrogen and GPER stimulated HIF1A and VEGFA expression and enhanced CRC cell proliferation and growth. In addition to regulation of hypoxia-related genes, estrogen acting through GPER potentiated hypoxia-induced migration of CRC cells, while under normoxic conditions estrogen suppressed cell migration of CRC cells *via* GPER (**Figure 5**).

Since an hypoxic tumor microenvironment is characteristic of advanced CRC tumors, and estrogen and hypoxia are functionally equivalent, it is tempting to speculate that this synergism can account for sexual dimorphism and poor survival with high GPER expression in late stage CRC female patients as was observed from Kaplan-Meier survival analysis. This hypothesis is supported by the observation that estrogen acting through GPER, down-regulates the expression of key DNA damage repair genes such as ataxia telangiectasia mutated (*ATM*) in CRC cells under hypoxic conditions (106). The deleterious effects of estrogen in hypoxia on ATM repression and HIF1A/VEGFA activation indicate that estrogen may also have pro-tumorigenic and pro-angiogenic effects deep within an hypoxic tumor. The cell-dependent estrogen effects of reducing cell growth and increasing apoptotic activity have previously only been reported in non-malignant colonocytes (young adult mouse colonocytes - YAMC) compared to isogenic p53 colonocytes (YAMC-Ras) (112). Since the loss of *ATM* expression is associated with poor survival in CRC (113), this finding identifies a potentially pro-tumorigenic role for estrogen in the hypoxic environment of ER-negative and GPER-expressing colorectal tumors.

**Figure 6:**
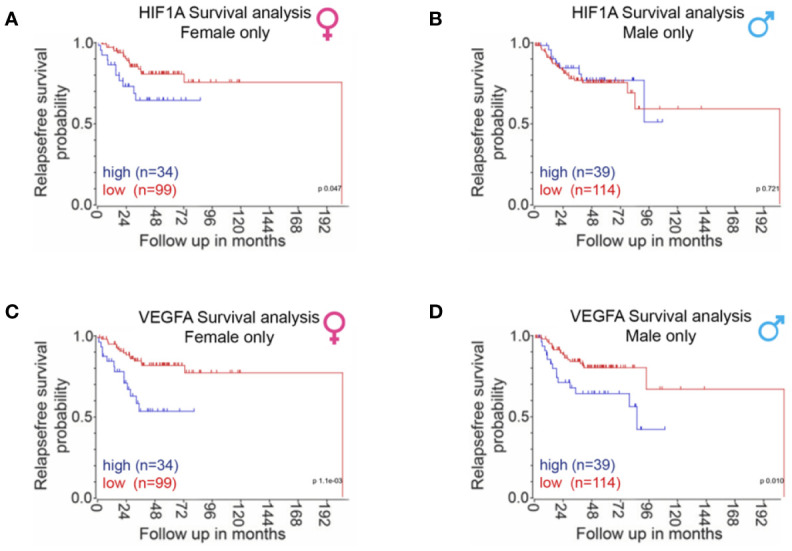
Sexual dimorphism of *HIF1A* and *VEGFA* expression in CRC survival. CRC patient survival analysis carried out using the R2 data visualisation platform from the Amsterdam Medical Centre R2: Genomics Analysis and Visualization Platform (http://r2.amc.nl) publically available datasets gse14333 + gse17538. The upper quartile of *HIF1A* gene expression was used to separate between high and low expression. P-values were calculated using a logrank test. Sufficient survival data was not available to calculate overall survival. **(A)** Relapse free survival in females only when associated with *HIF1A* expression, last quartile expression of *HIF1A* was associated with worse relapse-free survival in the female population (p = 0.047). **(B)** Relapse-free survival in males only when associated with *HIF1A* expression, last quartile expression of *HIF1A* was not associated with relapse free survival in the male population (p = 0.721). **(C)** Relapse-free survival in females only when associated with *VEGFA* expression, last quartile expression of *VEGFA* was associated with worse relapse-free survival in the female population (p = 1.1 e-3). **(D)** Relapse-free survival in males only when associated with *VEGFA* expression, last quartile expression of *VEGFA* was associated with worse relapse-free survival in the male population (p = 0.01).

Taken together, these observations would predict more deleterious effects of high *HIF1A* and *VEGFA* expression on CRC survival in females compared to males. This is observed to be the case in our interrogation of publically available CRC datasets; high expression of *HIF1A* was associated with worse relapse-free survival in the female CRC population but not in male patients (**Figures 6A, B**). The median expression also reflected this female sexual dichotomy of low survival with high HIF1A. High expression of *VEGFA* in the last quartile was also associated with poor survival in both sexes, the median expression, however, only showed statistical significance of high *VEGFA* expression with poor survival in females and not in males (**Figures 6C, D**).

It is thus important to bear in mind when assessing sexual dimorphism in CRC that the tissue microenvironment can modulate the action of estrogen and GPER in CRC malignancy and that the role of GPER in tumor progression differs depending on sex, tumor stage and microevironment. The sexual dichotomy of estrogen actions on CRC cell biology transduced through differential ER/GPER receptor expression under varying oxygen tensions may help resolve some of the controversies of epidemiological studies confounded by sex, age, HRT, and tumor stage in CRC.

### Metabolism and the Warburg Effect

There exists a wide body of literature on sex differences in metabolism in normal physiology and in metabolic diseases such as diabetes and obesity (114, 115), and in cancer (116). In cancer cells, the “Warburg effect” predominates such that energy is produced primarily *via* aerobic glycolysis in contrast to mitochondrial tricarboxylic acid cycle (TCA) and oxidative phosphorylation in non-cancerous cells (117, 118). This metabolic reprogramming to increase catabolic metabolism to produce ATP is important for cancer cell survival and proliferation especially under hypoxic and variable glucose conditions (119–121). As the Warburg effect takes place in the mitochondria, which are maternally inherited and show specific sexual dichotomy in function, the sex differences in cancer cell mitochondrial metabolic reprogramming might contribute to the sexual dimorphism in cancer susceptibility and outcome (122).

Sex differences in colon cancer metabolism are currently unknown but we can glean insights to be followed up in future CRC research from studies in brain and breast cancers. Estrogen promotes metabolic adaptation to rescue cell viability under hypoxic and low glucose conditions in breast cancer cells by supressing glycolysis and stimulating the TCA cycle *via* the up-regulation of pyruvate dehydrogenase (PDH) activity (123). This metabolic vulnerability was exploited by knockdown of PDH in the low-glucose state to potentiate ionizing radiation-induced apoptosis and reverse the cell survival effects of estrogen. Most metabolic effects of estrogens are mediated *via* ERα (124), however, little information exists on a role for estrogen receptors in modulating possible metabolic adaptations in colon cancer cells. In breast cancer cells, under low glucose conditions, estrogen stimulates PDH activity *via* AMPK phosphorylation that involves both ERα and ERβ (123). Since the expression of these ER receptor subtypes is lost in CRC tumor progression, it remains to be determined if estrogen and GPER can modulate sexual dimorphism in CRC cell survival through metabolic reprogramming under hypoxic conditions.

Lipid metabolism has also been shown to be altered in CRC and may play an important role in the development and progression of CRC (125). Abnormal lipid metabolism in CRC is characterised by elevated cholesterol, triacylglycerol and fatty acid biosynthesis (126) and sex differences in lipid metabolism are well established (127). These associations point to a possible sexual dimorphism in estrogen regulation of lipid metabolism, which may reduce CRC cell proliferation. This hypothesis is supported by the observation that estrogen decreases cholesterol, triglyceride and fatty acid biosynthesis in mouse liver (128). Interestingly, these metabolic effects of estrogen were only observed in the livers of mice expressing the membrane-localized ERα (MOER) and did not require nuclear ERα. Activation of MOER caused a rapid non-genomic AMP kinase phosphorylation of the sterol regulatory element-binding factor 1 (Srebf1), preventing its proteolytic cleavage by site-1 protease. Srebf1 was thus sequestered in the cytoplasm, reducing the expression of cholesterol synthesis-associated genes. These observations may point to a protective effect of estrogen to reduce colon cancer cell survival *via* reduced triglyceride biosynthesis, the main disturbed lipid marker of CRC progression (129).

### Drug Metabolism

Sex differences in drug metabolism are well-known but still remain under-appreciated in clinical trials. Sex hormones can affect the metabolism and pharmacokinetics of a wide range of toxic compounds and xenobiotics *via* genomic and non-genomic pathways affecting transcriptional regulation of the expression of drug metabolizing enzymes and modulation of metabolic signalling pathways (130). Sex differences in survival benefit from adjuvant chemotherapy in females has also been observed (131) and this protective effect is reinforced in females with CRC through transcriptional regulation of metabolism of xenobiotics by cytochrome p450 and of glutathione S-transferase, a key enzyme in the metabolism of toxic compounds and xenobiotics (132). Thus sexual dimorphism in the targeting of transcriptional factors regulating drug metabolism could be an additional feature of CRC survival to enhance chemotherapy effectiveness and reduce toxicity.

### Angiogenesis

We have seen that estrogen can modulate the expression of proliferative pathways such as VEGF in hypoxia *via* GPER signal transduction. Hypoxia (HIF1A) and VEGFA are pro-angiogenic leading to endothelial cell proliferation and formation of new blood capillaries to provide nutrients for tumor growth and metastasis (133). Estrogen has been reported to inhibit angiogenesis in a rat model of colon cancer by reducing the expression of VEGFA and HIFA (134). Sex differences in vascularization may be an intrinsic property of endothelial cells (ECs) as a consequence of female ECs expressing higher levels of eNOS (encoded by the *NOS3* gene) and cell adhesion integrins compared to male ECs (135). This sex difference would be expected to preferentially promote tumor angiogenesis and growth in female CRC.

Estrogen exerts rapid non-genomic vasodilatation and proliferative responses in vascular ECs (136). A myriad of signalling cascades affecting EC biology are rapidly (mins) activated by estrogen including cGMP, mitogen-activated protein kinases (MAPK), phosphatidylinositol 3-OH kinase (PI3K), and Akt, converging on the induction of endothelial nitric-oxide synthase (eNOS) (137), the latter a potent vasodilation signal. There is substantial evidence that both genomic and non-genomic estrogen actions in vascular ECs are mediated *via* plasmamembrane estrogen receptors. G-protein coupled receptors such as Gα(i) have been implicated in the rapid eNOS response to estrogen (138) and an N-terminus truncated ERα isoform, ER46, plays a key role in rapid endothelial responses to estrogen (139). Estrogen also exerts genomic effects in ECs, upregulating more than 250 genes as determined by DNA microarray analysis after 40 min exposure to hormone (140). One of the most important genes regulating EC migration, the cyclooxygenase-2 gene(*PTGS2*), showed strong upregulated expression by estrogen and rapid stimulated secretion of prostaglandins PGI2 and PGE2 *via* membrane-bound palmitoylated full-length ERα66. Both the genomic and non-genomic EC responses were prevented by inhibition of PI3K and Akt. Moreover, the estrogen-induced EC migration could be abrogated with inhibitors of cyclooxygenase-2 pointing to non-genomic regulation of the transcriptional events. The integration of membrane and nuclear estrogen receptor signalling appears to be an essential feature of estrogen induced proliferation and migration of vascular endothelial cells (141). An enhanced angiogenesis and vascular perfusion would be expected to translate into exacerbating effects of estrogen on tumor growth and metastases. Such an effect of estrogen on angiogenesis and tumor growth has been reported in breast and lung cancer, albeit correlated with nuclear ER expression (142). Currently it is not known if such sexual dimorphism in angiogenesis plays a role in CRC development although there is evidence that the non-genomic membrane ER effects of estrogen on EC biology do not translate into proliferative effects in uterine or breast cancers (143).

### Gut Microbiome

The impact of the gut microbiome on the development and progression of CRC is an area of growing interest, particularly over the last 50 years. The gut microbiome is located in close proximity to the colorectal epithelium and it is unsurprising that there is cross interaction. The role of the gut microbiota in CRC has been reviewed in detail elsewhere (144). The development of CRC has been associated with an overall reduction in microbial diversity (145) and specific enrichment of individual bacteria such as *Fusobacterium nucleatum* (146) and loss of potentially protective bacteria such as *Roseburia* (147). Dysregulation of the gut microbiome in inflammatory disease may affect CRC development as colonic tumorigenesis was observed to be attenuated by antibiotics in a mouse model of colitis induced CRC (148). These associative studies have generated many questions and much interest with regard to causative links between the microbiome and the generation of CRC and the potential of the microbiome to provide biomarkers or prognostic indicators for CRC.

With regard to sexual dimorphism, several studies in animal models have reported that estrogen signalling can help to maintain microbiome diversity. A 2016 study using multiple mouse strains demonstrated that ovariectomy led to microbial dysbiosis although this was impacted by both strain and diet (149). More specifically, dietary supplementation with the hormone estradiol has been shown to increase microbial diversity in healthy male mice and to impact the ratio of bacteria in the microbiome of a CRC-induced mouse model (150). The potential protective effect of estrogen was examined in a recent study using the AOM/DSS mouse model of intestinal-specific ERβ deletion and induced colitis and CRC. There is also evidence that ERβ expression may influence gut microbiome diversity in mouse models of colitis and CRC to attenuate these diseases (68). This study showed a reduction in gut microbiota diversity with the development of CRC, which was exacerbated in the absence of ERβ (151).

The gut microbiome has also been shown to affect estrogen bioavailabity. Indeed, the term estrobolome has been coined to refer to the gut microbiota with capacity to metabolise estrogens. The secretion of β-glucuronidase can deconjugate estrogens into their active forms (152). Presumably, gut bacteria expressing steroid sulfatase activity could alter the enterohepatic cycling of estrogenic compounds and estrogen metabolism (153). A decrease in gut microbiota may disturb this process and lead to a decrease in circulating estrogens to alter the progression of colorectal carcinogenesis.

Immune responses to inflammation as precursors to colorectal cancer may also be affected by the gut microbiome, particularly as a consequence of lifestyle factors and behaviours such as smoking and alcohol excess known to cause dysbiosis (154). These complex interactions between microbiome and immunity have been recently reviewed in-depth (155). Sex differences undoubtedly impact on the microbiome and teasing apart the contributions of diet, lifestyle choices versus genuine sexual dimorphism is notoriously challenging, especially with something as spatially and temporally unique as the gut microbiome. As researchers look to understand the gut microbiome in the protection against CRC and its treatment, it is important that sexual dichotomy in the microbiome is not overlooked.

## Sexual Dimorphism of Ion Channel Function in CRC

Estrogens are known regulators of epithelial ion channels and changes in ion transporter expression and activity are among the many epigenetic alterations found in colorectal cancer. Many studies show a close relationship between K^+^ channel expression and cancer progression, supporting a pivotal role for these channels in cancer development and as potential molecular targets or diagnostic tools. In particular, the role of voltage-gated K^+^ (Kv) channels has been proposed in various types of cancers, including CRC (156). Among this vast class of ion channels, one in particular stands out, the KCNQ1:KCNE3 potassium channel, which has been shown to be regulated by estrogen to modulate Cl^-^ secretion in female colon. KCNQ1 also controls the invasive phenotype of embryonic stem cells suggesting its role in tumorigenesis. The identification of the role of the KCNQ1:KCNE3 complex in colorectal cancer has been a major advance in our understanding of molecular mechanisms underpinning sexual dimorphism in CRC tumorigenesis and is discussed in detail in this section. To fully comprehend the role of estrogen-regulated ion channels in CRC we must first understand their function in the sexual dimorphism of estrogen actions on colon physiology

### Colorectal Cancer and Electrolyte Transport

The development of colorectal cancer is determined by multiple factors, which also control major physiological functions of the intestinal epithelium such as mucosal barrier protection and ion transport. Electrolyte handling and epithelial repair dysfunction in CRC patients are associated with a worsening outcome, influencing quality of life, tolerance to anticancer drugs, and conditioning survival (157, 158). Altered water and electrolyte transport affect the intestinal epithelium differentiation/proliferation balance and tumor development. During intestinal development, the organization of the epithelial mucosa depends on the establishment of net transepithelial NaCl secretion. Factors that regulate ion transport, in particular the activity of the Na+/K+ ATPase pump, also affect morphogenesis and tumorigenesis of the epithelium (159). An imbalance between intestinal Na^+^ absorption and Cl^-^ secretion participates in the process of tumorigenesis and is observed in the early stages of carcinogenesis (160). Moreover, Cl^-^ hypersecretion associated with chronic diarrhoea constitutes a risk factor for colorectal cancer (161).

### Estrogen Regulation of Colonic Epithelial Ion Transport

Estrogen plays an important role in regulating whole body fluid balance through modulation of water and ion secretion by the intestinal epithelium during the menstrual cycle (162). Rapid non-genomic and latent genomic physiological responses to estrogen occur in the colon, which affect epithelial ion secretion and absorption (163). The prominence of secretory and absorptive epithelia among the list of estrogen target tissues (kidney,lung,intestine, sweat gland) suggests an important role for estrogen in the regulation of whole body extracellular fluid balance through the modulation of secretory and absorptive ion transport processes. Over the course of the estrous cycle, large fluctuations in circulatory estrogen concentrations are experienced by the female body and these changes modulate the structure and function of reproductive tissues, particularly the uterus to facilitate embryo implantation. Thus the ion transport function of the colon is synchronized with the female reproductive cycle. The physiological role of the estrogen/estrous cycle-dependent, anti-secretory response in the colon may be to protect against fluid loss during the implantation window and promote endometrial/stromal expansion for blastocyst implantation (163).

Excessive dysregulated Cl^-^ secretion from the intestinal crypt is the underlying mechanism of secretory diarrhea in several etiologies of inflammatory bowel disease, such as ulcerative colitis and Crohn’s disease, which can predispose to CRC (164). Cl^-^ secretion across the colonic epithelium is two-step process involving Cl^-^ entry from the blood side into the cells *via* a basolateral Na^+^-K^+^-2Cl^-^ cotransporter (NKCC1) and exit of Cl^-^ into the intestinal lumen across the apical membrane of the cells *via* Cl^-^ channels (CFTR and CACC). As in all secretory epithelia, the ion channels and transporters of the colonic epithelial cells must operate in concert to achieve net Cl^-^ secretion. K^+^ channels localized in the basolateral cell membranes provide the electrical driving force for Cl^-^ efflux across the luminal membrane and blockade of specific basolateral K^+^ conductances will inhibit the Cl^-^ secretory process and are a therapeutic target to treat acute episodes of diarrhea. In the colon, the Kv7.1 channel (KCNQ1) is the primary K^+^ channel involved in maintaining cAMP-activated Cl^-^ secretion (165, 166). KCNQ1 forms hetero-multimeric channels with KCNE3 and is localized in the basolateral membrane to drive cAMP-activated Cl^-^ secretion in the small and large intestine, by recycling K^+^ that is transported into the cell by the basolateral NKCC1 and Na^+^-K^+^-ATPase. The association of the KCNE3 regulatory subunit with the pore-forming KCNQ1 channel complex locks the channel open at resting membrane potentials and confers cAMP sensitivity on channel conductance (167). The KCNQ1:KCNE3 channel K^+^ current causes membrane depolarization, which generates the electrical driving force required to maintain Cl^-^ secretion.

The active estrogen, 17β-estradiol, has been shown to inhibit KCNQ1:KCNE3 channels and reduce colonic Cl^-^ secretion under basal conditions (168) or when induced by cholera toxin and heat-stable enterotoxin (169), two of the major causes of secretory diarrhea in humans. The anti-secretory response to estrogen displays sexual dimorphism and has been observed in female but not in male rats (170). The inhibition of secretion by 17β-estradiol appears to be a general feature in Cl^-^ secretory epithelia and was reproduced in human bronchial epithelium (171). Moreover, the phytoestrogen, berberine, produces an anti-secretory effect in a human colonic adenocarcinoma cell line *via* inhibition of KCNQ1 channels (172). Many of the ion transporters targeted in the anti-secretory response to estrogen, such as CFTR and Kv channels, have a functional role in CRC development. Thus the anti-secretory action of estrogen in the colon may provide additional protection against CRC development in the female.

The molecular mechanism by which estrogen produces a sexual dimorphic anti-secretory response in female colon has been demonstrated (173). Estrogen-induces a rapid non-genomic PKCδ dependent phosphorylation of KCNE3 at residue serine82 causing the KCNQ1:KCNE3 channel complex to uncouple, thus reducing the channel conductance and thereby collapsing the electrical driving force for Cl^-^ secretion. Estrogen also induces a Rab-dependent endocytosis of KCNQ1 to maintain the anti-secretory response (174). The anti-secretory response to estrogen is maintained over several hours and its potency depends on the stage of the estrous cycle with maximal anti-secretory responses observed when circulating levels of estrogen are highest during proestrus and estrus (175). This would imply a genomic component of the latent long-lasting anti-secretory effects of estrogen underpinned by changes in the transcription and expression of ion transporters to sustain the anti-secretory response over days. The expression levels and degree of coupling between KCNQ1:KCNE3 were found not only to exhibit sexual dimorphism but also to be estrous cycle-dependent. Colonic crypts from male rats showed higher KCNE3 expression and >2-fold higher association of KCNE3 with KCNQ1 than found in female colon (170). This sex difference in channel subunit expression and association confers a sustainable genomic control by estrogen of KCNQ1:KCNE3 function such that estrogen downregulates KCNE3 expression and coupling to KCNQ1 and increases the relative proportion of free homomeric KCNQ1 (173). We shall later see that homomeric KCNQ1 can associate with β-catenin (encoded by *CTNNB1* gene) in CRC cells to prevent its nuclear translocation and impede cell proliferation and de-differentiation into a mesenchymal phenotype. Thus understanding the molecular mechanisms by which estrogen regulates KCNQ1:KCNE3 physiological function can reveal novel insights into sexual dimorphism in CRC.

### Estrogen Regulation of Kv Channels in CRC

Since voltage-gated K^+^ channels determine the membrane potential necessary for net Cl^-^ and Na^+^ transepithelial transport, these proteins constitute a central regulatory entity and variations in their expression and/or activity have major consequences on intestinal epithelial physiology, pathophysiology and development (176). Over the last twenty years, the evidence for K^+^ channel involvement in the development and growth of tumors has greatly expanded. While Kv channels are mainly expressed in excitable cells, these channels are also expressed in tumor biopsies and intestinal cell lines derived from colorectal cancers and their role in tumorigenesis has been proposed (177). In addition, the expression and activity of specific Kv channels have shown a significant correlation with the tumor malignancy grade (178). The literature clearly demonstrates that Kv channels are among targets of interest in the fight against cancer (179, 180) and the specific role of each Kv channel in tumorigenesis and the molecular mechanisms involved are being elucidated (181). This is notably the case of the estrogen-regulated KCNQ1 channel in colorectal cancer. KCNQ1 expression is associated with enhanced disease-free survival in CRC stages II, III, and IV disease (182). KCNQ1 plays a role in cell proliferation and participates in several stages of embryonic development (183). KCNQ1 expression is increased in germ cells and testicular tumors (184) and confers a proliferative invasive phenotype to embryonic stem cells (185). KCNQ1 is also an imprinted gene and an abnormal genomic imprinting is the cause of multiple neoplasias. A loss of imprinting of KCNQ1 has been described in colorectal cancer (186). A study using a transposon approach to identify genes that predisposed to colon cancer revealed KCNQ1 as the 3rd highest hit (187). Moreover, KCNQ1:KCNE3 is found in a diverse range of epithelial tissues including the kidney, pituitary, stomach, small intestine, liver, thymus, exocrine pancreas, ovarian tissue, and uterus that display sex differences in tumorigenesis (188). These observations point to a potential association of KCNQ1 expression and biological actions in colon cancer development. Up until recently, the functional role of the KCNQ1 channel and possible sexual dimorphism in colorectal cancer were unknown.

KCNQ1 is a tumor suppressor in CRC and its sustained expression has been linked to suppression of the Wnt/β-catenin signaling pathway that contributes to CRC tumor progression (189). KCNQ1 regulation may represent a link between the normal physiological actions of estrogen in the colon and the hormone’s apparent tumor-suppressive effects in CRC development. It is interesting to note that the expression of all of the major ion transporters involved in colonic transepithelial Cl^-^ secretion show sexual dimorphism and are modulated by estrogen and the estrous cycle in the female (170, 175), including CFTR a known tumor suppressor (190). Estrogen actions on KCNQ1:KCNE3 coupling are critical to hormone regulation of the channel function, sub-cellular location and surface expression. Estrogen activates a PKCδ dependent inhibition of KCNQ1 function by two main mechanisms: a rapid decrease in channel current caused by dissociation of KCNQ1 and KCNE3, and a reduction in current density due to internalization of the complex (173, 174). The decrease in current due to dissociation of KCNE3 from KCNQ1 is dependent on PKC phosphorylation of KCNE3 at residue Ser^82^. The putative estrogen regulated KCNE3 phosphorylation site Ser^82^ is conserved across all five KCNEs, giving rise to potentially an even wider range of homologous binding sites with a similar mode of sexual dimorphism action. A structural biology approach in combination with computational modelling has confirmed the structural basis for how estrogen-stimulated phosphorylation of KCNE3 Ser^82^ results in the dissociation of KCNE3 from the KCNQ1 channel pore and its potential role in the sexual dimorphism in CRC (191). This raises the exciting possibility that phosphorylation of Ser^82^ may mediate the effect of estrogen on the interaction of KCNE3 and KCNQ1 in CRC. This same phosphorylation site also modifies the behaviour of KCNE3/Kv3.4 channels (192), which are hypoxia-regulated Kv channels involved in cancer cell migration and invasion (193), opening up the possibility of a general KCNE3 mediated sex difference in cancer.

### KCNQ1 and Wnt/β-Catenin Signaling in CRC

**Figure 7:**
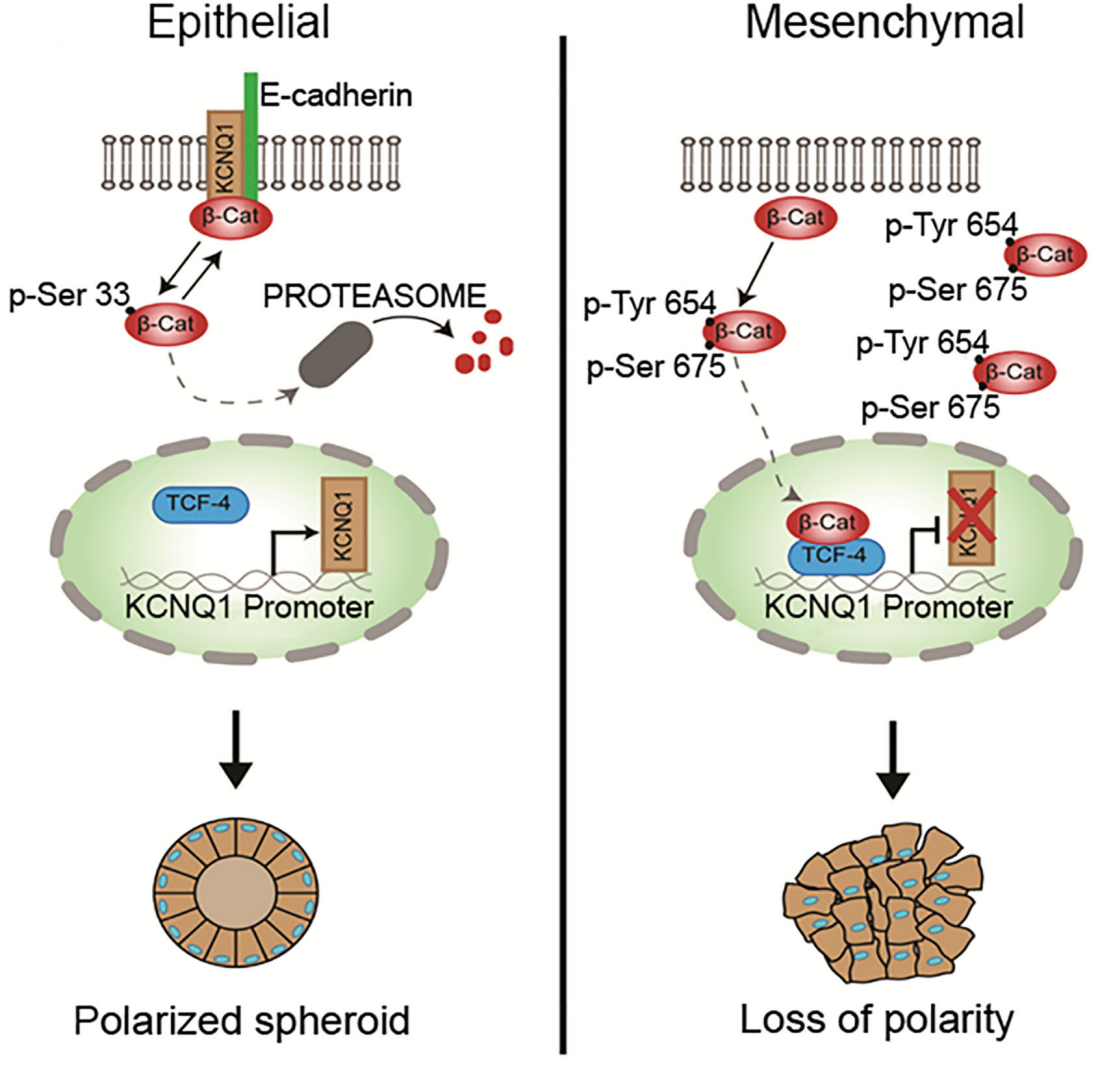
Regulation of epithelial mesenchymal transition *via* β-catenin:KCNQ1 interactions in CRC cells. In well-differentiated CRC cell lines, KCNQ1 stabilizes β-catenin with E-cadherin at adherens junctions to maintain a polarised epithelial phenotype. KCNQ1 expression also modulates the β-catenin phosphorylation state. KCNQ1 promotes Ser33 β-catenin phosphorylation, which reduces β-catenin stability by enhancing its proteosomal degradation. In poorly differentiated CRC cells in which Wnt/β-catenin activation is high, KCNQ1 expression is repressed by the β-catenin/TCF4 transcription factor. The low expression of KCNQ1 increases β-catenin phosphorylation at residues Tyr654 and Ser675, which activate nuclear translocation of activated β-catenin to stimulate the expression of proliferative genes and promote EMT and loss of cell polarity. In addition, phosphorylation at Tyr654 reduces β-catenin binding to E-cadherin and P-Ser675 protects β-catenin from proteosomal degradation [from (199)].

Although the KCNQ1 gene has been identified as a tumor suppressor in CRC tissues, the precise functional and molecular events linking KCNQ1 and CRC progression remain unclear. One obvious pathway that may interact with KCNQ1 is Wnt/β-catenin signaling, which plays a key role in intestinal homeostasis and is hyper-activated in early CRC tumorigenesis (194, 195). Deregulation of the Wnt/β-catenin signaling axis is present in more than 80% of CRCs. This can lead to β-catenin accumulation in the cytosol, increased nuclear translocation of activated β-catenin, interactions with members of the T-cell factor (TCF4) transcription factor family, and stimulation of β-catenin-dependent gene expression leading to increased cell proliferation and growth (196). The role of Wnt/β-catenin signaling in the development of colorectal cancer is now well-recognised (197) and a functional interaction between estrogen signaling and Wnt/β-catenin pathways has been reported in human colon cancer cells (198). Recent studies have shown convergence between KCNQ1 and β-catenin regulatory pathways in CRC and the molecular mechanisms regulating KCNQ1:β-catenin interactions and their effects on CRC cell differentiation, proliferation, and invasion (199). A negative feedback transcriptional loop was demonstrated between KCNQ1 and β-catenin expression, which regulates epithelial mesenchymal transition (EMT) in CRC cells (**Figure 7**). KCNQ1 expression was shown to suppress EMT in a wide range of CRC cell lines of varying stages of differentiation. Sustained expression of KCNQ1 protein was linked to suppression of the Wnt/β-catenin signaling pathway and the expression of KCNQ1 was lost with increasing mesenchymal phenotype in poorly differentiated CRC cell lines as a consequence of repression of the KCNQ1 promoter by β-catenin:TCF4. The high expression of KCNQ1 observed in well-differentiated CRC cells and its locking of β-catenin with E-cadherin (encoded by *CDH1* gene) into adherens junctions serves two purposes to inhibit or delay EMT and cell proliferation by promoting an epithelial phenotype and preventing nuclear transcription of β-catenin targeted proliferative genes, loss of cell polarity and progression to EMT. There is evidence that the cadherin-bound pool of β-catenin is generally much more abundant than the cytosolic fraction stabilized by Wnt such that the cadherins harbor a pool of β-catenin used for nuclear signaling (200). Moreover, it appears that Wnt must be activated to observe effects of E-cadherin expression on β-catenin release to the nucleus and cadherin-bound β-catenin was shown to feed into the Wnt nuclear transcriptional pathway (201). Of interest, the long non-coding RNA *KCNQ1OT1*, which down-regulates KCNQ1 expression has been shown to be activated by β-catenin and associated with poor patient survival in CRC (202). These observations suggest that estrogen-regulated KCNQ1 suppresses CRC cell proliferation and is a target gene of the Wnt/βcatenin pathway (203).

What could be the molecular mechanism for this protective action of KCNQ1 against EMT in CRC cells? One hypothesis is that high expression of homomeric KCNQ1 can serve as a molecular ‘bait’ to retrieve activated β-catenin from the cytosol into adherens junctions to preserve a well-differentiated epithelial phenotype. The estrogen-induced release of KCNQ1 from the KCNQ1:KCNE3 complex may enhance tumor suppressor effects of KCNQ1. It is possible that the estrogen action to free KCNQ1 from its association with KCNE3 and send it into an endocytic recycling pathway allows homomeric KCNQ1 to ‘fish’ for β-catenin in the cytosol and retrieve it into adherens junctions where it is locked in with E-cadherin. β-catenin has been shown to bind to homology units in K^+^ channels and regulate surface expression of the ion channel (204). The cycle of endocytotic retrieval of KCNQ1: β-catenin into adherens junctions could serve a dual purpose of inhibiting EMT and cell proliferation by promoting a well-differentiated epithelial phenotype and preventing nuclear transcription of Wnt/β-catenin targeted proliferative genes.

### Clinical Significance and Sexual Dimorphism of KCNQ1:KCNE3 in CRC

**Figure 8:**
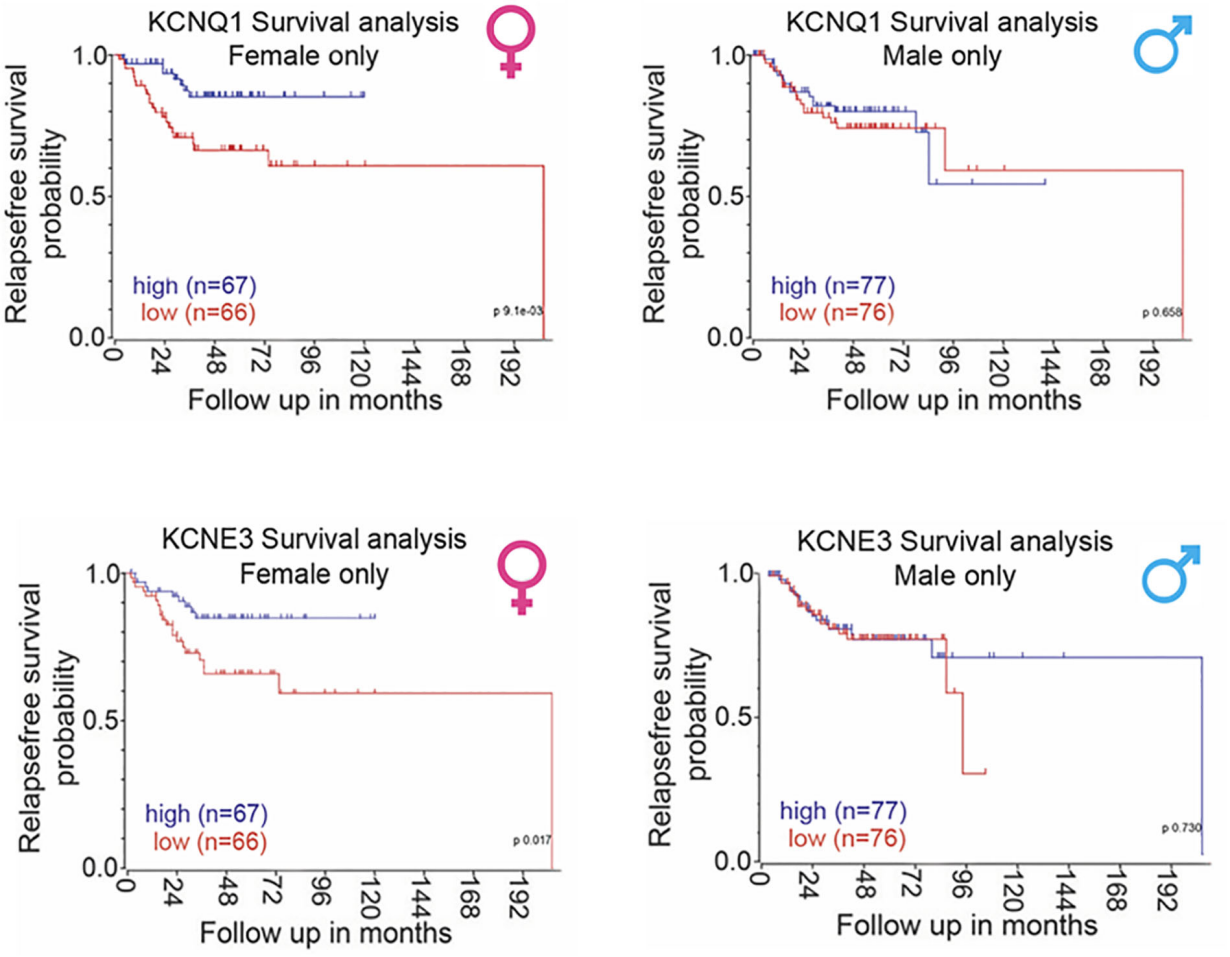
Sexual dimorphism in the correlation between *KCNQ1:KCNE3* and CRC patient relapse-free survival. Sex-differentiated analysis of human primary CRC tumor patient databases (accession number: GSE39582) showed a positive correlation between *KCNQ1* and *KCNE3* expression and disease-free survival in female patients only with no correlation observed in male patients. Survival analysis carried out using the R2 data visualisation platform from the Amsterdam Medical Centre R2: Genomics Analysis and Visualization Platform (http://r2.amc.nl) publically available datasets gse39582. Median gene expression values was used to separate between high and low expression. P-values were calculated based on a logrank test. Sufficient data was not available to calculate overall survival.

Bi-directional interactions between KCNQ1 and β-catenin regulate CRC cell differentiation processes and tumorigenesis (199). If KCNQ1:KCNE3 expression contributes to the protective female sexual dimorphism in CRC then we would expect this to translate into a positive correlation between *KCNQ1:KCNE3* expression and relapse-free survival in female but not in male CRC patients. Our Kaplan-Meier analysis of public–accessible CRC microarray databases (https://www.ncbi.nlm.nih.gov/geo/query/acc.cgi?acc=GSE39582), show that low *KCNQ1* and *KCNE3* expression are associated with poor relapse-free survival in patients with CRC and high expression of these Kv channel subunits correlate with better survival only in the female (**Figure 8**). The ensemble of molecular and clinical observations demonstrate that KCNQ1 is a target gene and regulator of the Wnt/β-catenin pathway and its repression leads to CRC cell proliferation, EMT, tumorigenesis and poor patient survival. The bi-directional interaction between β-catenin and estrogen-regulated KCNQ1:KCNE3 provides novel insights into the molecular mechanisms of sexual dimorphism in CRC.

### CFTR

Chloride ion channel function has also been associated with CRC development, the most notable being the cystic fibrosis transmembrane conductance regulator CFTR in which *CFTR* gene mutations are associated with causing cystic fibrosis (CF) (205). CFTR is a tumor suppressor and downregulated expression of CFTR is linked to CRC not only in CF patients but in the general population (206). A study, in the United States, of cancer risk in cystic fibrosis patients showed that they not only have a high risk (6-fold) of developing GI cancer but also a high risk of developing lymphoid leukaemia and testicular cancer (207). Moreover, endoscopic screening studies of adult CF patients found that polyps in individuals with CF appeared earlier, and were larger and more aggressive than those in the non-CF population (208, 209). Since then, the guidelines for endoscopic screening of CF patients have been modified and CF has been declared a hereditary colon cancer syndrome by the Cystic Fibrosis Foundation (210). A study of 90 Stage II CRC patients stratified by tumor *CFTR* expression showed disease-free survival at 3 years in the 25% of patients with lowest *CFTR* expression was 30% lower than those with higher expression (190). In a second cohort, CFTR protein expression was lower in tumor vs. normal tissue and *CFTR* mRNA expression was reduced in metastatic vs. non-metastatic tumors (211).

CFTR expression is highest at the base of the crypt and its expression has been reported to be involved in intestinal lineage differentiation. Thus, CFTR is posited to influence the renewal process of the intestinal epithelium and loss of CFTR expression may directly influence cancer initiating cells progression. In support of this hypothesis, *CFTR* deficiency was associated with increased clonogenicity and proliferation in murine colon and small intestinal organoids. In these studies *CFTR* expression was found to be localised in the *leucine rich repeat containing G protein-coupled receptor 5-positive* (LGR5+) stem cell (212). These findings support a role for *CFTR* in the environment of the cancer initiating cell or the stem cell itself. Furthermore, it has been shown that genetic suppression of *CFTR* colonic crypts resulted in stem cell proliferation and increased Wnt/β-catenin signaling and its functional expression in the active intestinal stem cell (ISC) population. The cell signaling mechanism involved increases in pHi in ISCs, which stabilizes the plasma membrane association of the Wnt transducer Dvl, likely facilitating Wnt/β-catenin signaling. Also, mice carrying an intestinal-specific conditional knockout of *CFTR* alone (Apc wildtype) showed enhanced nuclear localization of β-catenin, an indicator of activation, along with an elevated expression of Wnt/β-catenin target genes such as Cyclin d1 (Ccnd1), *Lgr5*, and the cluster of differentiation 44 (*CD44*) (190).

Further mouse genetic studies also demonstrated the functional significance of *CFTR* deficiency in colon cancer. *CFTR* was identified in the top 10% to 50% of candidate genes in three SB screens to identify CRC driver genes in *Apc* wildtype, *Apc*-deficient, and *TGF beta*-deficient (213). Moreover, CFTR expression was downregulated in CRC and promoter methylation may be responsible for this downregulation. The *CFTR* promoter methylation status is inversely associated with human CRC and is significantly correlated with lymph node metastasis and may be a potential marker for lymph node metastasis of CRC (214). The expression of *CFTR* in early stage human CRC patients stratified by risk of recurrence shows that loss of expression of CFTR is significantly associated with poor disease-free survival (215). In addition, there is evidence that CFTR expression (mRNA, protein) and function can be repressed by HIF1A in hypoxic epithelium (216), which is common in CRC, and most epithelial cancers. However, this response coupled with mitchondrial dysfunction and high oxidative stress in CRC cells requires further analysis to understand the role of CFTR in metabolic perturbations associated with CRC development.

Overexpression of CFTR suppresses CRC tumor growth by inhibiting the proliferation, migration, and invasion of CRC cells (214, 215). These effects are possibly related to CFTR function as a protective protein in the formation and stability of the cytoskeleton and cell-cell junctions. The physical interaction between the CFTR–PDZ binding domain (CFTR–PDZBD) and the PDZ domain of NHERF1 (Na^+^/H^+^ exchange regulatory cofactor 1) and SLC9A3R1 (solute carrier family 9, subfamily A, member 3 regulator) protect the organization of the actin cytoskeleton and tight junctions (217). In addition, loss of CFTR could disrupt tight junctions by preventing its interaction with TJP1 (tight junction protein 1) and ZO-1 (zona occludin 1), a key protein in both adherens and tight junction assembly and structure (218). CFTR protein–protein interactions may also mediate tumor suppression by acting as a signaling hub with metastasis suppressor proteins of the NM23 (non-metastatic clone 23)/NDPK (nucleotide diphosphate kinase) family that have been proposed to negatively regulate the Ras/PI3K(phosphoinositide 3 kinase) signaling pathway (219). Thus CFTR dysregulation at many levels of expression and function can disrupt colonic homeostasis and alter the tissue microenvironment to favour CRC development.

## Genetic Sexual Differentiation in CRC

Sex differences in colorectal cancer may not be entirely due to endocrine differences alone since age-dependent changes in estrogen or HRT do not always match the incidence and progression of the disease. Such hormone-independent sexual dichotomy in CRC may arise through sexual differentiation in genetic and epigenetic regulation of proteins controlling cellular growth and proliferation, immune responses and metabolism. There is strong evidence that differentially expressed genes and proteins may play an important role in sex differences in CRC (220).

As previously mentioned, right-sided (proximal) tumors occur predominantly in women and older patients, and are less common than left-sided distal tumors (23, 24). Proximal tumors have higher microsatellite instability, hypermethylation and mutation burden, whereas more distal tumors are characterized by chromosomal instability (221, 222). The burden of genetic mutations also varies between male and female CRC patients. Several reports have shown *BRAF*, *KRAS*, *PIK3CA* and *PTEN* are more mutated in right-sided tumors, whereas *APC* and *TP53* show higher mutation rates in left-sided tumors, even if they are ranked top in both sides (223). Interestingly, *BRAF* mutations, particularly *BRAF* V600E, occur predominantly in females and have been associated with poor prognosis (224). There is some evidence that the oncogene *KRAS* shows an age-dependent sex difference with older male patients carrying a higher number of *KRAS* mutations than older female CRC patients, though the numbers studied remain small (225). Male and female CRC patients show significantly different frequency of *KRAS* mutations in MSI-high tumors with high and low mutation burden (226). Also, *KRAS* Q61K missense mutation has been associated with the female gender (227). The fact that women tend to have more right-sided colon cancers, and *KRAS* is more frequently mutated in these cancers, could make the interpretation of sex differences in *KRAS* more complex. *EGFR* (HER-1 or erbB-1) is the most important therapeutic target in CRC patients in the absence of *KRAS/NRAS* mutations (228) and sex differences in CRC survival have been shown for specific germline polymorphisms within the *EGFR* gene (229). It is relevant to note that estrogen can cause trans-activation of EGFR *via* a non-genomic pathway acting through the membrane ER mERα to drive cell proliferation in many cancer types (230). Sexual dimorphism in oncogene expression and mutations has an important effect in differential CRC development (231). Molecular analyses found the oncogene *STK11* (also known as LKB1) to be more frequently mutated in males than in females (22.8% vs. 11.3%) (232). The *STK11* gene plays an important role as a tumor suppressor by activating AMPK to inhibit the AKT signaling pathway and its genetic silencing in CRC cell lines reduced invasion and metastatic potential. The sex-bias in *STK11* mutation frequency may have clinical repercussions to predict patient sensitivity to mTOR and SRC inhibitors.

The β-catenin gene (*CTNNB1*) is a well-known driver gene of CRC (233) and the oncogenic role of aberrantly activated β-catenin in colonic epithelial cells is supported by several studies (234). Over-activation of the Wnt signaling pathway drives the nuclear accumulation of β-catenin promotes the transcription of many oncogenes such as *MYC* and *CCND1* (235). In addition, different *CTNNB1* mutations could differentially affect the sensitivity to inhibitors of WNT affecting the sexual dimorphism of clinical outcomes in CRC patients (236).

Recent studies suggest that the proteomes of colorectal cancer may differ between males and females. Over-expression of certain miRNAs has been reported to be gender-specific in circulating miRNAs and colon tumor tissue from CRC patients (237). In a female mouse model of CRC, deficiency in miRNA-10a expression has been shown to increase colonic tumorigenesis acting in synergy with activated Wnt signaling (238). Other miRNA families also show sex differences in expression divergence such as the miR-10 family and miR-34 family, both implicated in CRC development (239). A promising sex-differentiated biomarker in DNA methylation panels is miR-137, which inhibits cell proliferation, migration, and invasion in lung adenomas and CRC  (240, 241), possibly through negative regulation of TCF4 expression and promoter methylation. Epigenetic factors such as DNA methylation may also play important roles in CRC gene expression, cell differentiation and genetic imprinting (242) and DNA hypomethylation has been associated with increased cancer malignancy (243). The genes regulating the circadian rhythm may also contribute to the better prognosis in females with colon cancer. Polymorphisms in the *CLOCK* sequence and the expression levels of miRNAs regulating the clock-genes were associated with longer overall survival of females with metastatic colorectal cancer compared to males (244).

A number of genetic syndromes have been associated with increased CRC risk. The most frequent of these are Lynch syndrome (mutations in DNA mismatch repair genes) and familial adenomatous polyposis or FAP (*APC* mutations). Combined, these syndromes account for only 5% of all CRC cases. Lynch syndrome/hereditary non-polyposis colorectal cancer (HPNCC) and familial adenomatous polyposis (FAP), have been reported to present a significant gender gap in associated CRC risk (245). With Lynch syndrome/HPNCC females have a cumulative CRC lifetime risk of 30%, whereas men have a risk of 54%. On the other hand, FAP patients exhibit desmoids with sex-specific growth characteristics and a differing response to medical therapy. It is unclear whether gender differences or true sexual dimorphism underlie the difference in CRC incidence between men and women born with these syndromes. Further work is needed to clarify the interacting effects and the relative contributions of sex-biased gene expression signatures to tumorigenesis in CRC.

## Sexual Dimorphism of the Immune System in CRC

The immune system shows marked sex differences both in terms of gender (behavioral, nutritional, microbiome, and environmental adaptations) (246) and in true sexual dimorphism (endocrine and genetic differences) (247). There is strong evidence that sex hormones are pivotal in determining the strength of immune responses between the sexes (248) and females benefit in combating acute inflammatory infections possibly through XX chromosome advantages to suppress inflammatory cytokines (249). The double X chromosome confers an expression gain to females for immune responsive gene adaptation, whereas the male Y chromosome reduces the capacity for immunogenic gene expression (250). The sex differences regarding the activation of certain X-linked genes involved in innate immunity and the modulation of immune responses by sex hormones (251) point to potential advantages/disadvantages for CRC cells to evade immune destruction between the sexes The ability of females to mount a more robust immunological response to acute inflammation (252) may be protective in the early stages of innate immune responses in CRC, however, chronic inflammation in advanced tumor progression may compromise the adaptive immune system in females and exacerbate tumor progression.

Chronic mucosal inflammation has been implicated in neoplastic transformation of the healthy colon in inflammatory disease such as ulcerative colitis and Crohn’s disease (253, 254) and inflammatory bowel disease can be a precursor of CRC with worse prognosis (255). Previous studies have shown that epigenetic and microRNA factors may also be involved in conferring an X-linked female advantage in immunity to acute infection (256) and recent studies in mice and humans indicate that extra X genes may give females an immunological advantage (257). An overactive immune response, however, can be a double-edged sword. In healthy females, the inflammatory response is greater than in males. This can be an advantage to females to fight acute infection but could be detrimental in cases of chronic inflammatory disease in CRC patients. To complicate things further, a recent study suggests that the paternal X chromosomes passed to a daughter have higher levels of X inactivation, which, in the case of the immune system, can dampen the expression of certain genes, possibly promoting a pro-inflammatory response in females (258). There is a salient lesson here to avoid over-simplification of sex differences associated with innate and adaptive immune responses.

Estrogen is a well-documented regulator of inflammatory responses (259). Sex differences in adaptive-immune related genes expressed in T cells and estrogen modulation of IFN-γ and of CD3^+^ and CD8^+^ T cell invasion in CRC tumors suggest a stronger adaptive inflammatory and cytotoxic T cell response in females (260). On the other hand, growing evidence related to the CRC microenvironment and high levels of intra-tumoral neutrophils have been associated with unfavorable recurrence-free survival (261). Moreover, Tumor-Associated Neutrophils (TAN) play a key role in the acquisition of a malignant phenotype and are an independent factor for poor prognosis of patients with CRC (262). Estrogen modulation of the tumor immune microenvironment displays sexual dimorphism in reducing myeloid cell invasion of liver metastases associated with colon carcinoma (263). This protective effect of estrogen on tumor immunogenicity may be transduced *via* ERβ as re-expression of ERβ in CRC cell lines caused strong down-regulation of IL-6 inflammatory signaling networks (264). The IL-6/JAK2/STAT3 pathway has been shown to exert stimulatory actions on myeloid cell to promote colon tumorigenesis (265). Given the complexity and plasticity of tumor-immune cell interactions in CRC and their regulation by estrogen and X-linked genes, any useful analysis of sexual dimorphism in immune adaptation in CRC tumorigenesis necessitates the use of multi-parametric analyses (266).

Currently, immunotherapies are often used for the treatment, remission, and even possible cure of several cancers. There are diverse approved immunotherapy options for CRC including ones for tumors that are microsatellite instability-high (MSI-H) or mismatch repair deficient (dMMR) (267). A recent clinical trial (nivolumab plus ipilimumab cohort of CheckMate-142) has demonstrated the long-term efficacy of immunotherapy in MSI-H and DMMR CRC (268). The scientific evidence overwhelmingly shows that females and males differ in the outcomes following use of immunotherapies for the treatment of cancer (269), however, not enough consideration has been given to sex and gender differences in the adverse reactions, efficacy, and uptake of biologics, including those that impact the immune system (270). Without a sex- and gender-sensitive approach to immune treatments, disparities in outcomes will persist (271). These considerations are highly relevant to sex differences in immunotherapies in CRC, which primarily target the adaptive immune system in particular, when the sexual dimorphism of cancer immunotherapy is still a nascent field (272).

## Gender Gap in CRC

CRC rates can vary markedly around the world due to lifestyle associated risks and the availability of population-screening programmes for early detection. These environmental, socio-economic and lifestyle choices underpin a gender gap in CRC as distinct from sexual dimorphism of biological origin. However, biological sex differences can also contribute to the impact of environmental and lifestyle choices on the development of CRC.

### Diet and Lifestyle

Multiple lifestyle factors combine with biological sex differences to contribute to disparities between the sexes in many diseases including cancer (273). Women have generally healthier dietary habits than men, with higher fibre and lower meat consumption, and less alcohol intake. Diet, exercise and other lifestyle factors have previously been associated with CRC risk (274). Recently, it has been observed that lifestyle factors such as smoking and diet could be linked with specific molecular CRC subtypes (275). Some of the challenges in reproducing clinical and epidemiological findings, or to reach significance in population-based cancer studies, are due to heterogeneity amongst patient groups with regard to multiple confounding factors such as lifestyle, diet, gut microbiome, immunity, and tumor characteristics. Hence, studies incorporating the newer integrative discipline of Molecular Pathological Epidemiology (276), in which the study population is segregated by similar molecular pathological characteristics, should include biological sex-differences, gender and behaviour delineated risk factors to assess treatment response and prognosis in CRC.

The impact of lifestyle factors on CRC risk seems to differ in men and women implying an underlying biological contribution. The European prospective investigation into cancer and nutrition study (EPIC) has reported that a high inflammatory profile (proinflammatory diet+ sedentarism +obesity) showed a strong association with higher risk for colon cancer in men (hazard ratio of 2.11 [95% CI, 1.50–2.97]) and no significant association in women (277). A meta-analysis including 4 cohort and 7 case-control studies determined that soy consumption (a phytoestrogen) reduced by 21% the risk for CRC in women and was not associated with CRC risk in men (278). Obesity has been described to increase the risk for hormone-driven cancers (279). Although mechanistic associations between obesity and CRC remain unclear, hyperinsulinemia and hyperleptinemia may partially explain the adiposity associated with CRC progression in postmenopausal women (280). Adipose tissue becomes an important source of estrogens in postmenopausal women and obese men. Higher Body Mass Index and lower physical activity levels have been positively associated with higher levels of circulating estrogens in postmenopausal women (281).

### Bowel Screening

Gender differences in uptake of bowel screening have been noted with women tending to consider CRC as a male disease and less likely to benefit from promotional screening interventions (282), whereas men tend to procrastinate the task more than women (283). Non-invasive faecal occult-blood tests and colonoscopies are the gold standard for CRC screening as they facilitate early detection of asymptomatic CRC and the removal of the precancerous adenomas (284). Five year-survival rates for early stage colorectal cancer are close to 90%, whereas survival rates drop to 15% at advanced stages. Population screening strategies established by developed countries have had a major survival benefit by detecting a higher proportion of localised, early stage malignancies. In many developed countries, a population screening programme has been established to screen the at-risk population. “BowelScreen” is the Irish example of such a programme and aims to screen every man and woman aged 60–69 years old for a home faecal immunochemical test (FIT) every 2 years (285). As capacity grows, many countries are expanding their programs to make screening available for all the population from 55 to 79 years old. Younger adults with increased risk for CRC due to family history or a genetic condition are eligible for regular colonoscopies.

### Age and Comorbidity

Age is the predominant risk factor for CRC. Currently, 80% of colon cancer patients and 75% of rectal cancer patients are diagnosed above the age of 60 (286). As mentioned above, certain hereditary syndromes, such as Lynch syndrome and FAP, are associated with higher risk of developing CRC but that risk displays a significant gender gap. Lynch syndrome/HPNCC females have a cumulative CRC lifetime risk of 30%, whereas men have a risk of 54% (245). Based on this evidence, it is timely to suggest not only gene but sex-based adapted screening and surgical recommendations for both syndromes. A gene-sex-based recommendation predicated on penetrance should be the target of large international studies such as the International Mismatch Repair Consortium (IMRC) or the Mallorca Group in order to stratify screening recommendations (287).

Recent data collected in North America, Australia, China, and Western Europe show colon cancer incidence is increasing rapidly among young adults under 50 years old (288). Most of early-onset CRC cases lack any family history or known genetic condition, which excludes them from early prevention screenings (289, 290). This trend has been associated with recent changes in lifestyle regarding a poor diet and/or lack of physical activity (291). Age apart, there are many environmental factors that have been shown to increase CRC incidence. The “western diet” with high red and processed meat, low fibre intake, unhealthy habits such as smoking or excessive alcohol consumption, sedentary life style, obesity, and diabetes mellitus have all been associated with a higher CRC risk (292). From the current epidemiologic data, it is expected by the year 2040 there will be around 2 million new cases and 1 million deaths due to CRC (15). Even with comparable lifestyle choices, it appears that women maintain a higher level of protection than men against CRC, implicating sexual dimorphism as a key element in future research and therapeutic strategies (293).

## Conclusions

**Figure 9:**
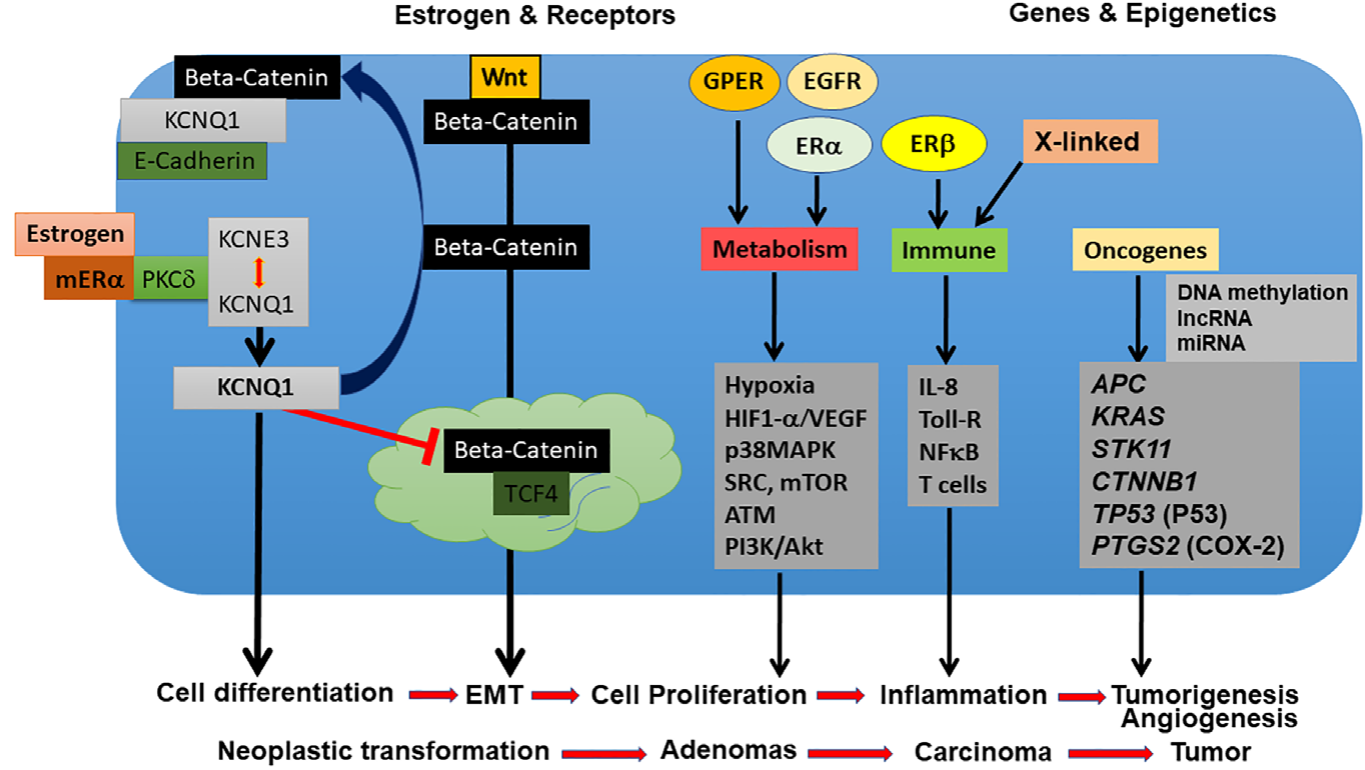
Sexual dimorphism targets of estrogen signaling, receptors and genes underpinning sex differences in the development of CRC. Estrogen, receptors and genes modulate ion channels, metabolism, innate and adaptive immunity, and oncogene expression to activate non-genomic and genomic responses underpinning CRC development from neoplastic transformation, adenomas, carcinoma to tumor.

A growing body of work indicates there can be striking sex differences in the incidence and development of colon cancer. Sexual dimorphism in the biology of gene and protein expression, and in endocrine cellular signaling, underpins the sexual dichotomy in colon cancer as summarised in **Figure 9**. The observed short‐term non-genomic regulation and long‐term genomic regulation of colon cancer development by estrogen and X-linked genes raises exciting new possibilities for precision medicine and molecular targeted therapeutics. Sex differences in non-reproductive organ cancers are complex, but fundamental to our nature. A deeper understanding of the molecular mechanisms of sex bias in cancers is essential and timely in the age of precision medicine.

This review underscores the potential wealth of information and novel signaling pathways that may be uncovered by studying sexual dimorphism in colon cancer. It is clear that estrogen plays a significant role in protecting women against CRC. Recent advances in the field have substantially enhanced our understanding of the molecular mechanisms underlying the sexual dichotomy in CRC though many questions still remain, such as how long the protection from estrogen lasts and what other molecular and lifestyle mechanisms are at play, particularly in older women. Elucidating the circumstances under, which estrogen changes from a protective role to a more neutral or even pro-tumorigenic role in CRC is a clinically-relevant research area ripe for exploration.

## Author Contributions

MA and BH wrote the manuscript. MA, VB, HH, and BH drew the figures. HH analyzed the public accessible databases shown in **Figures 6** and **8**. BH secured funding for this work. All authors contributed to the article and approved the submitted version.

## Funding

This work was supported by funding from the Higher Education Authority of Ireland Programme for Research in Third Level Institutions PRTLI Cycle 5 to BH. HH was supported by a PhD scholarship from the Health Research Board Ireland and MA by a PhD scholarship from private donor DOCTRID Ireland.

## Conflict of Interest

The authors declare that the research was conducted in the absence of any commercial or financial relationships that could be construed as a potential conflict of interest.
